# On the epitaxial growth in ALD Co_3_O_4_- and NiO-based bilayers†

**DOI:** 10.1039/d5nr01212k

**Published:** 2025-04-14

**Authors:** Renée T. M. van Limpt, Cristian A. A. van Helvoirt, Mariadriana Creatore, Marcel A. Verheijen

**Affiliations:** a Eindhoven University of Technology 5600 MB Eindhoven The Netherlands r.t.m.v.limpt@tue.nl m.a.verheijen@tue.nl; b Eindhoven Institute of Renewable Energy Systems (EIRES) 5600 MB Eindhoven The Netherlands; c Eurofins Materials Science Netherlands 5656 AE Eindhoven The Netherlands

## Abstract

NiO and Co_3_O_4_ are versatile materials studied for a plethora of applications, yet their performance for a specific application relies on the control of their crystallographic texture and corresponding surface facets. Achieving such control can be challenging, often requiring hetero-epitaxial growth on single-crystalline substrates, which are frequently incompatible with the requirements of the application. The combination of NiO and Co_3_O_4_ in heterostructures provides potential to control texture due to their similar crystal structures, whilst retaining the possibility to work with more versatile substrates. In this study, atomic layer deposited (ALD) thin films based on cyclopentadienyl precursors and an oxygen plasma are adopted to tailor the crystallographic texture of NiO from 〈100〉 to 〈111〉 using an ALD Co_3_O_4_ template layer, and similarly, to modify the Co_3_O_4_ texture from 〈111〉 to 〈100〉 on a NiO template. The films are shown to conform to the crystal orientation of the template material, whilst crystallizing directly in their own stable crystal structure with corresponding metal atom coordination. Further investigation includes ALD process parameters for NiO growth: the film texture is found to depend on the choice of co-reactant and the above-highlighted hetero-epitaxial relationship is stronger for plasma-based processes. In conclusion, these results demonstrate an original approach for application-oriented crystallographic engineering in thin films.

## Abstract 2

The work shows that atomic layer deposited hetero-stacks enables controlling the crystalline texture. This provides the opportunity of tuning the atomic arrangement at the surface of thin films on templates relevant for practical applications.

## Graphical abstract

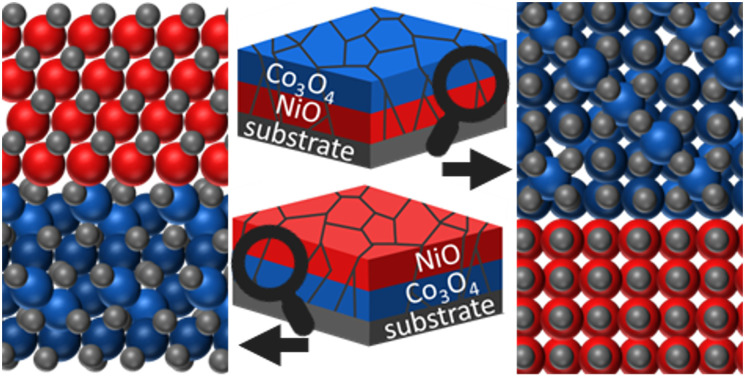

The chemistries of nickel oxide (NiO) and cobalt oxide (Co_3_O_4_) are versatile and widely studied for their implementation in various applications such as solar cells,^1–3^ supercapacitors,^4–10^ gas sensors,^11–14^ batteries,^15–22^ and (electro-)catalysis.^23–29^ A key property influencing the performance of these materials for a specific application is the orientation of the crystals within the film and the type of surface facet correlated to it.^30–37^ The NiO (111) facet is, for example, preferred for gas sensors,^38^ whilst NiO (110) is more efficient for ethane activation.^39^ Similarly, Co_3_O_4_ (111) is preferred for Li–O_2_ batteries,^31,40^ whilst the (110) surface is preferred for photothermal methanol oxidation.^41^ In the context of oxygen evolution reaction (OER) electrocatalysts in water electrolysis, the optimal facets are Co_3_O_4_ (100) and NiO (110). Co_3_O_4_ (100) exhibits higher activity due to the formation of a thicker active (oxy)hydroxide skin layer as compared to Co_3_O_4_ (111), possibly due to differences in metal coordination and/or defects.^42–44^ Likewise, NiO (110) facets show superior activity by stabilizing the β-Ni(OH)_2_ form, as opposed to the less active α-Ni(OH)_2_ form.^45^

Controlling the crystallographic texture of a NiO or Co_3_O_4_ film is challenging. Films often lack a preferred growth orientation when deposited on amorphous substrates,^46–60^ necessitating hetero-epitaxial growth on crystalline substrates such as *c*-Al_2_O_3_ (0001)^61–66^ or MgO (100)^57,63,64,67^ to achieve oriented growth.^55,64,68–70^ However, these substrates are often not compatible with the targeted applications.

The cubic crystal structures of NiO and Co_3_O_4_ differ in the coordination number of their metal atoms. Interestingly, the interplanar distances in several directions of the two crystal lattices are quite compatible; the Co_3_O_4_ lattice has ∼4% smaller interplanar distances compared to equivalent spacings in NiO with similar atomic positions of metal atoms.^71,72^ This close alignment enables hetero-epitaxy,^73^ providing an opportunity to obtain preferred growth orientations for Co_3_O_4_ and NiO that cannot be obtained by growing single layers, but may be realized by continuing the texture from the underlying layer.

By combining NiO and Co_3_O_4_ in heterostructures, composites and core–shell structures have already been shown to synergistically modulate their chemical and electronic band structures, benefiting applications such as gas sensors, batteries, and electrocatalysis, among others.^73–80^ Zhang *et al.*^73^ demonstrated that the NiO–Co_3_O_4_ interface is metallic, significantly enhancing conductivity and charge transfer during the OER as compared to NiO and Co_3_O_4._ This heterointerface was therefore identified as the primary driver for improved performance as an OER catalyst. These results suggest that NiO and Co_3_O_4_ film stacks could also possibly improve performance in various applications. In the context of electrocatalysis, Co_3_O_4_ could act as an active, stable, and protective base layer, while a NiO coating could be utilized to increase the active surface area.^26,73,81–84^

Successful implementation of hetero-stacks requires a discrete, well-defined interface between the oxide layers. In this work, atomic layer deposition (ALD) is adopted; it can be expected to provide such an interface because it is based on sequential self-limiting surface reactions. This self-limiting nature originates from strong adsorption between the selected precursor and anchoring groups (*e.g.* hydroxyl) on the underlaying surface. Furthermore, ALD provides an opportunity to work with complex, large surface area substrates that are, for example, required for catalysis. The deposition of NiO using ALD has been successfully demonstrated with various precursors and co-reactants for a range of applications. For example, Koushik *et al.*^1^ deposited NiO using Ni(^Me^Cp)_2_ and O_2_ plasma for application in solar cells, while Chung *et al.*^85^ adopted Ni(dmamp)_2_ and H_2_O for non-volatile memory devices and Haghverdi Khamene *et al.*^27^ employed Ni(^*t*^Bu-MeAMD)_2_ and H_2_O for NiO serving as an oxygen evolution reaction electrocatalyst. Similarly, Co_3_O_4_ has been successfully deposited using CoCp_2_ and O_2_ plasma to fabricate anodes for batteries as reported by Donders *et al.*,^86^ while Nandi *et al.*^87^ employed Co_2_(CO)_8_ and O_3_ to synthesize Co_3_O_4_ as a catalyst in NaBH_4_ hydrolysis.

For templating purposes, a textured film is desired. Donders *et al.*^88^ have demonstrated a preferential (111) orientation for Co_3_O_4_ films based on CoCp_2_ and O_2_ plasma, whilst the combination of Ni(^Me^Cp)_2_ and O_2_ plasma has been shown^1,27^ to yield a preferential (200) orientation. These processes will therefore be employed to investigate whether the texture of Co_3_O_4_ and NiO can be tuned by synthesizing thin-film stacks. However, the crystallinity of ALD films and the texture, *i.e.*, the preferred crystallographic growth direction, are influenced by several factors, including the choice of reactant/co-reactant, deposition temperature, impurity level in the film, film thickness, and the choice of substrate.^89^ Following the demonstration of the hetero-epitaxial relationship between NiO and Co_3_O_4_, we will investigate the influence of key process parameters – including the temperature, reactant, and co-reactant – on the growth of NiO on Co_3_O_4_. This will help identify the optimal ALD process conditions required for achieving hetero-epitaxial growth.

## Results and discussion

As an initial step, thin films of the individual oxides on *c*-Si have been characterised. These films were deposited on *c*-Si (100) with 2.5 nm native oxide at a substrate table temperature of 300 °C using previously developed plasma-enhanced ALD processes based on Ni(^Me^Cp)_2_ as a precursor for NiO and CoCp_2_ as a precursor for Co_3_O_4_ and an O_2_ plasma co-reactant for both processes.^1,88,90^

**Fig. 1:**
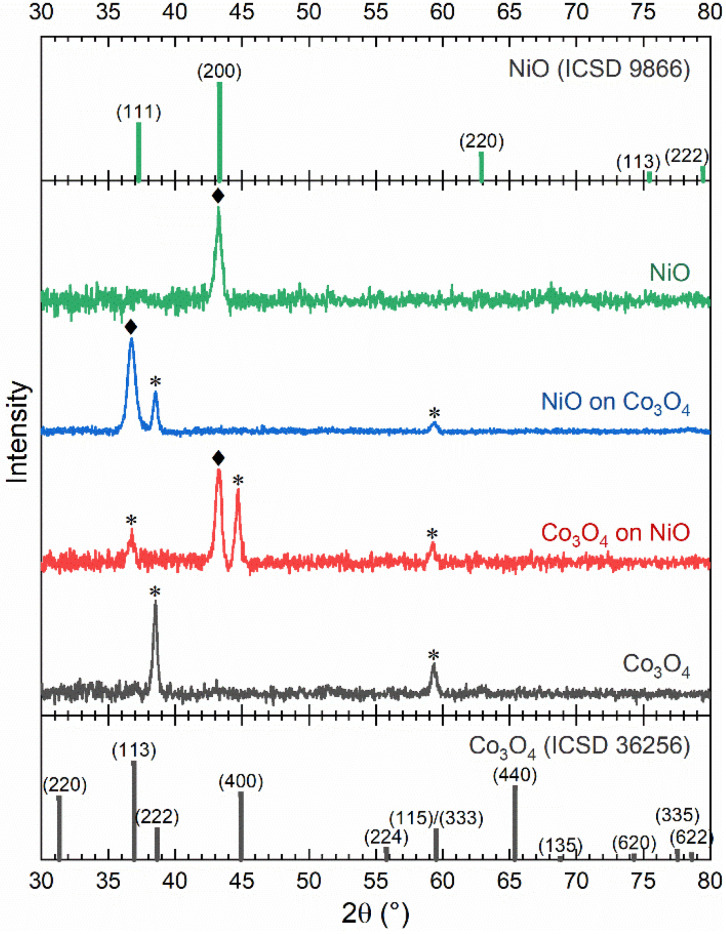
Goniometric X-ray diffractograms of the *c*-Si/NiO, *c*-Si/Co_3_O_4_, *c*-Si/NiO/Co_3_O_4_ and *c*-Si/Co_3_O_4_/NiO stacks. Peaks associated with the NiO rock-salt structure are indicated by ◆ and peaks associated with the spinel phase of Co_3_O_4_ are indicated by *. ICSD reference measurements of both NiO and Co_3_O_4_ are provided.

The crystal structures of the films were investigated using X-ray diffraction (XRD) (Fig. 1). Note that the ALD films are typically of polycrystalline nature. The 23 nm NiO film shows a prominent feature at 43.24 ± 0.04°, which is identified as the (200) reflection of the cubic *Fm3m* (225) rock-salt structure.^71^ No other diffraction peaks are observed, implying a strong 〈100〉 texture. The derived lattice constant of 4.18 ± 0.01 Å is in close agreement with the theoretical value for NiO of 4.17 Å (ICSD 9866). The XRD pattern of the 51 nm Co_3_O_4_ film displays two distinct features at 38.52 ± 0.04° and 59.36 ± 0.04°, which are identified as the (222) and (333) peaks of the cubic *Fd*3*m* (227) spinel structure.^72^ No other peaks are present, indicating a strong 〈111〉 texture for spinel Co_3_O_4_. It is important to note that this texture led to the assignment of the (333) reflection instead of the (115) reflection for the 59° feature. The extracted lattice constant for Co_3_O_4_, 8.09 ± 0.01 Å, again closely matches the literature value of 8.07 Å (ICSD 36256). Therefore, it can be concluded that both ALD films are polycrystalline under the investigated growth conditions, and have a strong texture.

Next, hetero-stacks of the oxides were deposited. The *c*-Si/53 nm Co_3_O_4_/27 nm NiO stack displays three distinct features in the diffractogram. The peaks at 38.54 ± 0.04° and 59.36 ± 0.04° correspond to those identified in the 〈111〉-textured Co_3_O_4_ template. The third peak at 36.74 ± 0.04° is attributed to the (111) peak of the NiO rock-salt structure. The sole presence of the NiO (111) peak indicates that the NiO layer adapts its preferred growth direction to follow the 〈111〉 texture of the underlying Co_3_O_4_ template. Rocking curve XRD analysis of the angular distribution of the Co_3_O_4_ (111) reflection yields an FWHM of the texture orientation of ∼6°. The texture of the NiO overlayer closely matches the distribution of the underlying Co_3_O_4_ (see the ESI†). The NiO (111) peak position corresponds to a lattice constant of 4.23 ± 0.01 Å, which is significantly larger than the lattice constant observed for NiO directly deposited on *c*-Si. This out-of-plane elongation of the unit cell is suggested as being attributable to compensation for the compressive strain within the horizontal plane, which facilitates lattice matching between the NiO and Co_3_O_4_ layers, as the lattice parameter of Co_3_O_4_ is 1.94 times larger than that of NiO, and equivalent interatomic distances of Co_3_O_4_ are 0.97 times those of NiO (Table S1†).

Conversely, four features are observed in the diffractogram of the *c*-Si/29 nm NiO/42 nm Co_3_O_4_ stack. The feature at 43.28 ± 0.04° is attributed to the (200) reflection of the NiO template. The dominant Co_3_O_4_ feature at 44.72 ± 0.04° corresponds to the (400) peak of the spinel phase, indicating that Co_3_O_4_ adjusts its preferred growth direction to align with the underlying 〈100〉-textured NiO template. However, additional features at 36.76 ± 0.04° and 59.28 ± 0.04°, attributed to the (113) and (115) reflections of the spinel structure, show the presence of secondary texture components. Furthermore, the lattice constant of 8.10 ± 0.01 Å, associated with the Co_3_O_4_ features, shows that the Co_3_O_4_ unit cell is not strained to match with that of NiO.

**Fig. 2:**
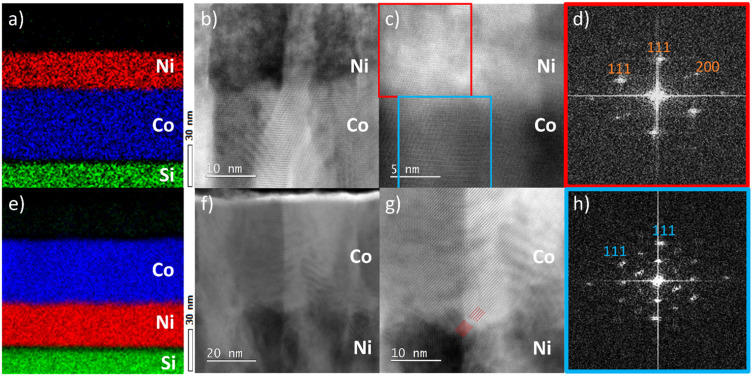
(a and e) EDX elemental mappings of the stacks. Complementary cross-sectional BF-STEM images of the (b and c) *c*-Si/Co_3_O_4_/NiO stack and the (f and g) *c*-Si/NiO/Co_3_O_4_ stack. (d and h) The corresponding 〈011〉 zone-axis patterns of (c). The {111} planes of both oxides are indicated in (g).

The crystal growth of the films was further investigated using cross-sectional scanning transmission electron microscopy (STEM) (Fig. 2). The STEM images have been acquired from selected grains that could be imaged along the 〈110〉 zone axis. These images are assumed representative of the whole film. Cross-sectional energy-dispersive X-ray spectroscopy (EDX) elemental mappings of both stacks confirm the deposition of well-defined, compositionally separated homogeneous films. Bright-field STEM images reveal that both Co_3_O_4_ and NiO grow in a columnar morphology, confirming the polycrystalline nature of the films. Co_3_O_4_ furthermore displays a larger lateral larger grain size compared to NiO. Based on the bright-field TEM contrast, a larger disorder is observed in the NiO crystals as compared to Co_3_O_4_ (see also Fig. S1 and S2†).

In both stacks, crystals are observed to continue their growth across the interface. The hetero-epitaxial relationship between the Co_3_O_4_ template and the NiO film is demonstrated in Fig. 2c and the corresponding fast Fourier transforms (FFT) from selected areas are shown in Fig. 2d and h. Both the FFT patterns are characteristic of 〈011〉 zone-axis patterns. Their identical orientation proves the identical crystallographic orientation of the domains at both sides of the interface. The denser pattern in Fig. 2h reflects the doubled unit cell dimensions of Co_3_O_4_ compared to NiO. In the atomic resolution image of Fig. 2c, the hetero-epitaxy relationship can also be recognized; horizontal {111} planes of both crystal structures are aligned parallel to the interface, while an additional set of {111} planes at an inclination of 71° continue across the interface. Similarly, detailed images of the *c*-Si/NiO/Co_3_O_4_ stack also reveal the hetero-epitaxial relationship between NiO and Co_3_O_4_ grains. This is highlighted in Fig. 2g, where the diagonally oriented {111} planes of NiO and Co_3_O_4_ run parallel. Based on the combined XRD and TEM results, we conclude that NiO grows epitaxially on Co_3_O_4_ likely in a compressively strained fashion to accommodate lattice mismatch between both layers. Similarly, Co_3_O_4_ predominantly grows epitaxially on NiO. However, no strain is observed in the XRD pattern, suggesting that, in this case, the epitaxial relationship is likely facilitated by misfit dislocations at the interface rather than by lattice strain.

**Fig. 3:**
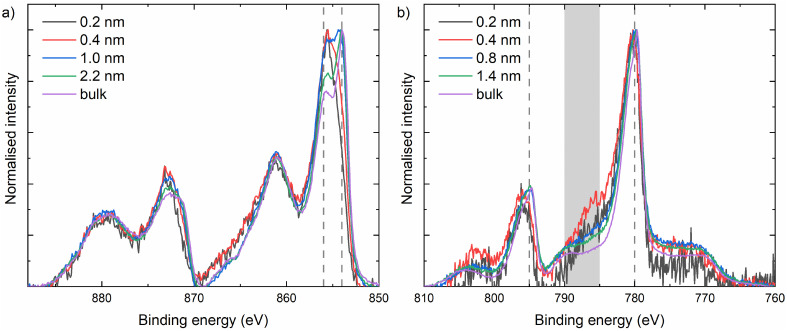
XPS measurements of films following 5, 10, 20 and 40 ALD cycles of (a) nickel oxide on *c*-Si/Co_3_O_4_ traced using the Ni 2p spectrum and (b) cobalt oxide deposited on *c*-Si/NiO traced using the Co 2p spectrum. The features at 854 eV and 856 eV are indicated in the Ni 2p spectrum. In the Co 2p spectrum, the 785–790 eV region is highlighted, whilst the dashed lines indicate the 15 eV spin–orbit split.

As is evident from the TEM results, rock-salt NiO grows on spinel Co_3_O_4_ and *vice versa*. From the STEM images, it cannot be concluded whether this phase transition occurs exactly at the interface between the two layers, as the interface between NiO and Co_3_O_4_ is not atomically flat in both stacks. Therefore, X-ray photoelectron spectroscopy (XPS) was employed to further investigate the NiO–Co_3_O_4_ interface (Fig. 3). Initial measurements of the single oxides on *c*-Si were performed for reference. NiO exhibits the characteristic 854 eV feature with a shoulder at 856 eV in the Ni 2p spectrum, indicative of the octahedrally coordinated Ni^2+^ states expected for its rock-salt structure.^26,90–94^ Co_3_O_4_ shows both the Co 2p_3/2_ feature at 779.7 eV and the characteristic spin–orbit splitting of 15.0 eV, which represents the mixed tetrahedral Co^2+^ and octahedral Co^3+^ states characteristic of the spinel structure.^26,90,93–96^

The interface between NiO and Co_3_O_4_ has been investigated by depositing layers of NiO from 5, 10, 20, and 40 ALD cycles on a Co_3_O_4_ film and *vice versa*. The thickness of the film layers deposited on the template increases with the number of ALD cycles and was calculated using the Thickogram model (see the ESI†). All Ni 2p spectra were corrected for the cobalt oxide Auger features, and all Co 2p spectra were corrected for the nickel oxide Auger features (see the ESI†). NiO growth on Co_3_O_4_ was monitored using the Ni 2p spectrum (Fig. 3a). Initially, the 856 eV shoulder is the dominant feature, which is indicative of the presence of Ni^3+^.^26,90–94^ The presence of Ni^3+^ is attributed to the NiO adapting to the underlying Co_3_O_4_, which also contains Co^3+^. This observation aligns well with previous work, where we showed that a supercycle ALD process of NiO and Co_3_O_4_ can form both NiCoO_2_ (rock-salt) and NiCo_2_O_4_ (spinel) films depending on the cycle ratio.^90^ However, after 20 ALD cycles (∼1.0 nm), the 854 eV shoulder becomes dominant again, and after 40 ALD cycles (∼2.2 nm), only a small increase in the 856 eV shoulder is observed compared to NiO on *c*-Si. It can therefore be concluded that the NiO film is affected by the underlying spinel only for the first few ALD cycles (∼0.5 nm) and returns to its thermodynamically stable valence state of +2 within the first nanometres of deposition.

Similarly, an increased loss feature is observed between 785 and 790 eV in the Co 2p spectrum at 5 and 10 ALD cycles of Co_3_O_4_ on NiO (0.2 and 0.4 nm, respectively). This increase in the loss feature is accompanied by an increased spin–orbit splitting of 15.9 and 15.7 eV, respectively, indicating the presence of octahedrally coordinated Co^2+^ states. However, after 20 ALD cycles (∼0.8 nm), the energy split is reduced to 15.2 eV and the loss feature has decreased in intensity, indicating rapid relaxation of Co_3_O_4_ in its spinel structure consisting of Co^2+^ and Co^3+^.^26,90,93–96^

Based on these observations, we can conclude that the crystallographic orientation of NiO can be tuned using a Co_3_O_4_ template, and *vice versa*, due to a hetero-epitaxial growth relationship between both materials. The two materials adopt the crystal orientation of the template material but crystallize (almost) directly in their own stable crystal structure, with the corresponding metal atom coordination.

**Fig. 4:**
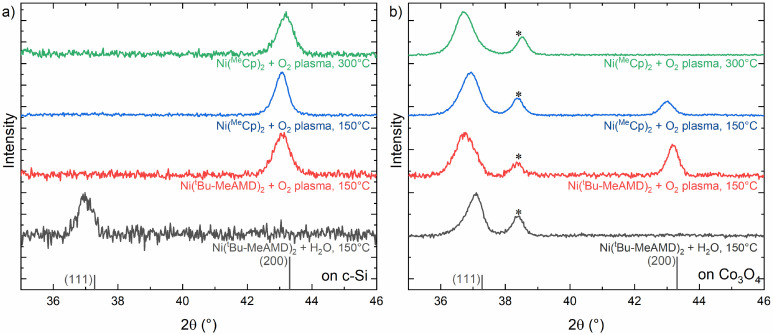
Goniometric X-ray diffraction patterns of NiO deposited using several ALD processes on (a) *c*-Si and (b) *c*-Si/Co_3_O_4_. The Co_3_O_4_ (111) peak is indicated by * and the ICSD database reference of NiO is provided (ICSD 9866).

As mentioned in the introduction, an ALD process is characterized by a range of parameters that can be tuned to influence the growth behaviour of the oxides. The most common process parameters, *i.e.*, choice of precursor, co-reactant, and temperature, were varied in this study to evaluate their impact on the epitaxial growth of NiO on Co_3_O_4_. The influence of ALD parameters on NiO deposited on *c*-Si/native oxide was investigated initially (Fig. 4a and S7†). First, the substrate table temperature was decreased from 300 °C to 150 °C. This caused a slight shift in the (200) XRD peak to lower 2*θ*, such that the lattice constant slightly increased to 4.20 ± 0.01 Å, suggesting the presence of strain. Next, the precursor was changed to Ni(^*t*^Bu-MeAMD)_2_, while maintaining the 150 °C substrate table temperature. No significant differences were observed between both precursors, as the film also grew in the 〈100〉 orientation. The strain observed in both films might be attributed to various factors such as differences in stoichiometry, thermal expansion coefficients, and texture-dependent minimization.^97^ At higher substrate temperatures, the strain might be reduced by higher surface mobilities of the adatoms during growth.^89^ Finally, the O_2_ plasma was replaced with H_2_O as a co-reactant in the Ni(^*t*^Bu-MeAMD)_2_-based process at a substrate table temperature of 150 °C. This shifted the texture of the film to 〈111〉 with a lattice parameter of 4.21 ± 0.01 Å. It is speculated that the change in the preferred growth direction originates from the presence of hydroxyl groups that stabilize the (111) surface.^98^ This is consistent with XPS measurements of the O 1s spectrum (Fig. S8†), which show an increase in the hydroxide shoulder only for the H_2_O-based process.

A comparison between the above-mentioned results and those related to NiO deposited on Co_3_O_4_ (Fig. 4b and S9†) reveals that the tunability of the NiO texture is influenced by the ALD parameters. At a lower deposition temperature (Ni(^Me^Cp)_2_ precursor, 150 °C), both the (111) and (200) peaks are observed, indicating an incomplete epitaxial relationship, although the (111) peak remains dominant. Lattice parameters of 4.21 ± 0.01 Å and 4.20 ± 0.01 Å are deduced from the (111) and (200) peaks, respectively, suggesting that the film remains strained, albeit to a lesser extent than that at 300 °C. Similarly, for the plasma-enhanced ALD process based on Ni(^*t*^Bu-MeAMD)_2_, both peaks are present, but the (200) peak is more dominant as compared to that of the Ni(^Me^Cp)_2_ film. Interestingly, a distinct difference in the lattice parameters is observed, with 4.23 ± 0.01 Å for the (111) reflection and 4.20 ± 0.01 Å for the (200) reflection. Here, it is important to note that in the goniometric XRD measurements, horizontal lattice planes are probed and the (111) peak originates from a different subset of crystals in the film than the (200) peak. This suggests that the 〈111〉-oriented grains grow epitaxially on the Co_3_O_4_ grains in a strained manner to compensate for the difference in lattice parameters between both structures, while the 〈200〉-oriented grains grow independently of the substrate. Additional measurements of a thinner NiO film reveal that the (200) peak is initially not observed (Fig. S10†). It is therefore hypothesized that the epitaxial relationship exists in the initial phase of growth, but the ALD process does not supply enough energy to fully retain the strained epitaxial layer, causing the 〈100〉-oriented growth preferred on *c*-Si to develop also in thicker NiO layers.

The NiO film based on Ni(^*t*^Bu-MeAMD)_2_ and H_2_O remains fully (111) oriented. However, a shift to higher 2*θ* is observed compared to the epitaxial (111) orientations of the plasma-based processes. The lattice parameter of 4.19 ± 0.01 Å shows reduced strain compared to deposition on *c*-Si, suggesting that there is no hetero-epitaxial relationship with the underlying Co_3_O_4_ template. Additional cross-sectional TEM measurements (Fig. S11†) confirm that NiO grains do not evolve as continuation of the Co_3_O_4_ grains and no epitaxial relationship exists at the interface. This might be related to the absence of the energetic ions generated by the plasma, which potentially stimulate ALD surface reactions. The increased reactivity provided by plasma species reduces the thermal energy required at the substrate to drive ALD surface chemistries and epitaxy.^99,100^

Additional XPS studies on the Co_3_O_4_–NiO interface were conducted for the several ALD NiO processes to investigate whether differences in epitaxial growth are related to the chemical environment. A comparison of the Ni 2p spectra of the NiO films (on *c*-Si) shows that all films exhibit the expected Ni^2+^ oxidation state. However, both films deposited using the Ni(^*t*^Bu-MeAMD)_2_ precursor show small amounts of nitrogen (∼1.5 at% N/(N + O + Ni)), irrespective of the co-reactant (Fig. S12†). This nitrogen in the H_2_O process is assigned to the nickel-bonded N in the surface amidinate moiety in line with the study of Zhao *et al.*,^101^ whilst the O_2_ plasma results in nitrate-like bonds.^101,102^ The presence of impurities is a known factor preventing crystallization and could therefore partially explain why less epitaxial growth is observed for the plasma process with Ni(^*t*^Bu-MeAMD)_2_ as compared to Ni(^Me^Cp)_2_, which has negligible impurity levels.^89,90,103^

**Fig. 5:**
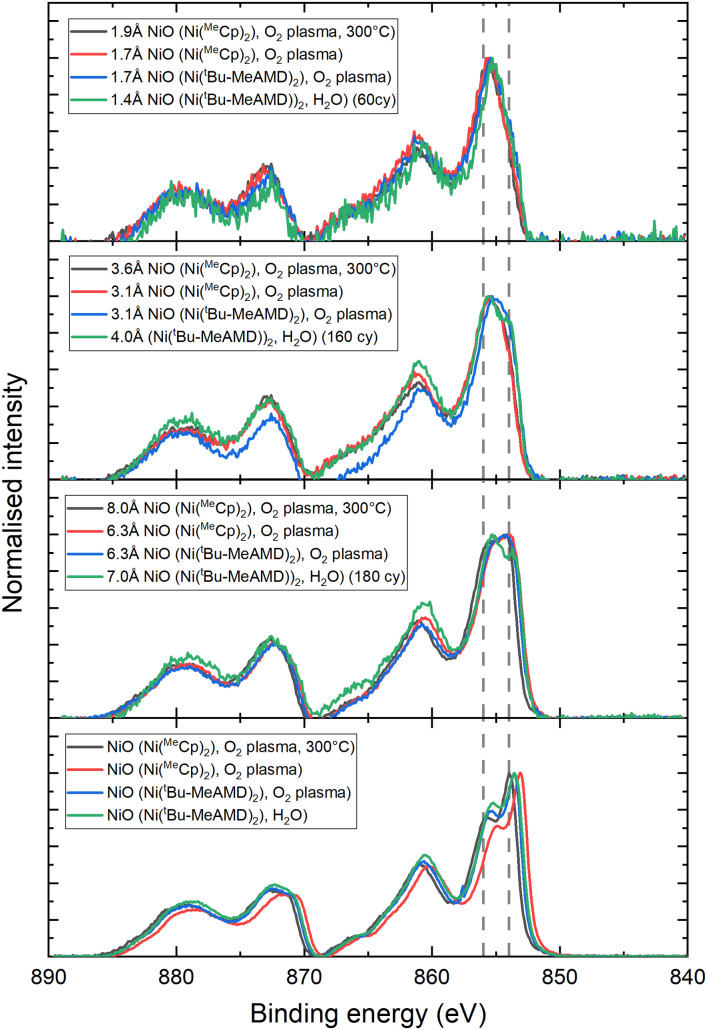
XPS spectra of various thicknesses of ALD NiO films on *c*-Si/Co_3_O_4_. The features at 854 eV and 856 eV are indicated as a guide to the eye.

Investigation of the nucleation of the NiO layers on Co_3_O_4_ reveals that all plasma-based processes show similar growth behaviour (Fig. 5). Initially, NiO nucleates with Ni^3+^ oxidation states, but transitions to the expected Ni^2+^-based film in the early stage of growth within the first 20 cycles. The H_2_O process, on the other hand, displays a different nucleation behaviour. Although the sub-monolayer growth is similar, a more defined peak splitting is observed between the main peak and shoulder for thicker films. This could indicate the formation of separate phases, possibly a nickel hydroxide phase, and therefore a more defective growth. Besides the different chemical environments, a major nucleation delay is observed for H_2_O-based NiO on Co_3_O_4_. While a nucleation delay of ∼40 cycles is common on *c*-Si, a delay of ∼160 cycles is observed on Co_3_O_4_ (Fig. S13†). This nucleation delay suggests a defect-driven initial growth as opposed to the epitaxial growth for all other NiO processes.

## Conclusions

In this study, we have combined NiO and Co_3_O_4_ in heterostructures using ALD to control their crystallographic texture. ALD films based on cyclopentadienyl precursors and an oxygen plasma exhibit strongly textured 〈100〉 rock-salt NiO and 〈111〉 spinel Co_3_O_4_ in a columnar morphology. In the heterostructure, Co_3_O_4_ adapts its growth direction to follow the 〈100〉 texture of NiO films. Similarly, NiO adapts its crystal orientation to mimic the 〈111〉-textured Co_3_O_4_. While Co_3_O_4_ retains its lattice constant of 8.10 Å when templated on NiO, NiO adapts its out-of-plane lattice constant from 4.18 Å to 4.23 Å to facilitate compressively strained epitaxy with the Co_3_O_4_ template. XPS reveals that both materials crystallize in their own stable crystal structure with corresponding metal atom coordination within the first 20 ALD cycles.

Variations in the ALD process parameters of NiO growth reveal that the NiO crystal orientation on *c*-Si is independent of the precursor or temperature but depends on the co-reactant. Switching from O_2_ plasma as co-reactant to H_2_O shifts the texture to 〈111〉. The epitaxial relationship in the stack, however, is more sensitive to ALD parameters. Lowering the substrate table temperatures from 300 °C to 150 °C leads to a partial loss of the 〈111〉 texture, such that 〈100〉-oriented grains develop when the film is thick enough. This effect is more pronounced for the Ni(^*t*^Bu-MeAMD)_2_-based films as compared to the Ni(^Me^Cp)_2_-based films, possibly due to nitrogen impurities in the film. No epitaxial growth is observed for the H_2_O-based process.

In conclusion, we have demonstrated that ALD hetero-stacks offer an effective approach for controlling crystalline texture. Placing our results in perspective, we infer that this method shows strong potential for achieving crystallographic control of thin films on templates relevant for practical applications. This work therefore reveals an additional merit of ALD beyond established benefits such as thickness control, precise composition tuning, and excellent uniformity and conformality. It expands the ALD toolbox to include crystallographic control, opening new opportunities for the design and engineering of thin films.

## Experimental methods

All NiO and Co_3_O_4_ films were deposited using a home-built plasma-enhanced ALD reactor described elsewhere.^104^ The system features an inductively coupled plasma source powered at 13.56 MHz. The pumping system consists of a turbomolecular pump paired with a rotary vane pump, capable of reaching a base pressure below 1 × 10^−6^ mbar. The reactors walls were heated to 100 °C, whilst the 4-inch wafer-based substrate holder temperature was varied between 300 °C and 150 °C, as indicated in the manuscript. Cobaltocene (CoCp_2_, 98% purity, ACROS Organics) was placed in a stainless steel cylindrical bubbler and heated to 80 °C and introduced into the chamber using an argon carrier gas through a line at 100 °C. Similarly, 1,1′-dimethylnickelocene (Ni(^Me^Cp)_2_, 97% purity, Sigma-Aldrich) was heated to 55 °C and delivered to the chamber using argon gas through a line at 75 °C. Both precursors reacted with a 100 W O_2_ plasma co-reactant. Both precursors were introduced using a 4 s/4 s/5 s precursor dosing/Ar purge/pump sequence with *x* s/1 s/3 s O_2_ plasma exposure/O_2_ purge/pump sequence, where *x* = 3 s for NiO deposition and *x* = 5 s for Co_3_O_4_ deposition.^1,26,88,90^ All films were deposited on *c*-Si (100) with 15 minutes of O_2_ plasma treatment before deposition. Hetero-stacks were deposited without breaking the vacuum to ensure a clean interface.

Additional NiO deposition processes were based on the bis(*N*,*N*′-di-*t*-butylacetamidinato)nickel precursor (Ni(^*t*^Bu-MeAMD)_2_, 99.999% purity, STREM) contained in a stainless steel cylindrical container heated to 90 °C and delivered using Ar through a line heated to 120 °C. In both processes, the precursor is delivered by 2× (3 s precursor dose + 2 s Ar line purge) followed by a 20 s pump. Afterwards, either an O_2_ plasma/O_2_ purge/purge of 5 s/1 s/10 s or a H_2_O/pump of 1 s/60 s was used. The thicknesses of the various NiO films are presented in Table 1.

**Table 1:** Thicknesses of the NiO films as presented in Fig. 4, S4, S6 and S7† as determined by spectroscopic ellipsometry

	On Co_3_O_4_	On *c*-Si
Ni(^Me^Cp)_2_ + O_2_ plasma, 300 °C	27 nm	23 nm
Ni(^Me^Cp)_2_ + O_2_ plasma, 150 °C	30 nm	40 nm
Ni(^*t*^Bu-MeAMD)_2_ + O_2_ plasma, 150 °C	31 nm	18 nm
Ni(^*t*^Bu-MeAMD)_2_ + H_2_O, 150 °C	19 nm	18 nm
Ni(^*t*^Bu-MeAMD)_2_ + O_2_ plasma, 150 °C (thin, see the ESI†)	11 nm	

The crystallography of the films was investigated using a Bruker Discover D8 (Cu K_α_ radiation, *λ*=1.54060 Å) in goniometric XRD mode at an axis offset of 3° to remove *c*-Si substrate peaks (Fig. S14†). Measurements in the 2*θ* range from 30.00–80.00° were performed with a step size of 0.04° at an integration time of 1 s, whilst measurements in the 2*θ* range from 34.00–46.00° were performed with a step size of 0.02° at an integration time of 4 s.

X-ray photoelectron spectroscopy measurements were performed using a Thermo Scientific KA 1066 spectrometer with monochromatic Al K_α_ X-rays. The binding axis was calibrated by the 248.8 eV adventitious C 1s peak. Thickness values were determined from the XPS data using a Thickogram model based on the Ni 3p (66.6 eV) and Co 3p (59.1 eV) features. The attenuation length was determined using the Tanuma–Powell–Penn-2M method, with a bandgap of 2 eV^105^ and a density of 5.0 g cm^−3^ for Co_3_O_4_. For NiO, a bandgap of 3.8 eV^1^ was used in combination with a density of 6.5 g cm^−3^ for plasma NiO and a density of 5.1 g cm^−3^ for thermal NiO^2^. Densities of NiO (plasma, Ni(^Me^Cp)_2_, 300 °C) and Co_3_O_4_ were determined by Rutherford backscattering spectrometry (RBS) (Table 2). RBS was carried out by Detect99 using a 200 keV He^+^ beam at perpendicular incidence and two detectors at scattering angles of 170° and 107°. Channel mode was used to reduce the background under the oxygen peaks. PIXE was used to distinguish Co from Ni. For PIXE, a 2.7 MeV H^+^ beam was applied at perpendicular incidence. The X-ray detector was positioned at an angle of 45° between the beam and the specimen normal, and 30 μm Kapton was used as an absorber. The PIXE spectra were analysed with the Gupix^106^ PIXE simulation package, and the resulting Co/Ni ratio was entered as a Co_*x*_Ni_*y*_ “molecule” in the WiNDF^107^ RBS simulation package to determine the absolute amounts. Electron microscopy images were acquired using a probe-corrected JEOL ARM 200F transmission electron microscope operated at 200 kV equipped with a 100 mm^2^ Centurio SDD EDX detector. Cross-sectional TEM samples were made using a focused ion beam (FIB) following a standard lift-out sample preparation protocol.

**Table 2:** Elemental concentrations of the films deposited on NiO and Co_3_O_4_ films after 40 ALD cycles as determined by RBS

Template	Template thickness (SE) (nm)	40-cycle film	Film thickness (XPS) (nm)	O (TFU)	Co (TFU)	Ni (TFU)	Density of the template (g cm^−3^)
Co_3_O_4_	35	NiO	1.4	182 ± 10	129 ± 3	8.0 ± 0.3	5.0 ± 0.3
NiO	21	Co_3_O_4_	2.2	128 ± 10	8.0 ± 0.3	106 ± 2.4	6.5 ± 0.6

## Data availability

The data that support the findings of this study are openly available in Zenodo at https://doi.org/10.5281/zenodo.14160831.

## Conflicts of interest

There are no conflicts to declare.

## Supplementary Material

NR-017-D5NR01212K-s001

## References

[cit1] Koushik D., Jošt M., Dučinskas A., Burgess C., Zardetto V., Weijtens C., Verheijen M. A., Kessels W. M. M., Albrecht S., Creatore M. (2019). Plasma-Assisted Atomic Layer Deposition of Nickel Oxide as Hole Transport Layer for Hybrid Perovskite Solar Cells. J. Mater. Chem. C.

[cit2] Phung N., Zhang D., van Helvoirt C., Verhage M., Verheijen M., Zardetto V., Bens F., Weijtens C. H. L., Geerligs L. J., Kessels W. M. M., Macco B., Creatore M. (2023). Atomic Layer Deposition of NiO Applied in a Monolithic Perovskite/PERC Tandem Cell. Sol. Energy Mater. Sol. Cells.

[cit3] Gibson E. A., Smeigh A. L., Le Pleux L., Fortage J., Boschloo G., Blart E., Pellegrin Y., Odobel F., Hagfeldt A., Hammarström L. (2009). A P–Type NiO–Based Dye–Sensitized Solar Cell with an Open–Circuit Voltage of 0.35 V. Angew. Chem..

[cit4] Xia X., Tu J., Zhang Y., Wang X., Gu C., Zhao X. B., Fan H. J. (2012). High-Quality Metal Oxide Core/Shell Nanowire Arrays on Conductive Substrates for Electrochemical Energy Storage. ACS Nano.

[cit5] Wang X., Hu A., Meng C., Wu C., Yang S., Hong X. (2020). Recent Advance in Co3O4 and Co3O4-Containing Electrode Materials for High-Performance Supercapacitors. Molecules.

[cit6] Dar F. I., Moonoosawmy K. R., Es-Souni M. (2013). Morphology and Property Control of NiO Nanostructures for Supercapacitor Applications. Nanoscale Res. Lett..

[cit7] Xu J., Gao L., Cao J., Wang W., Chen Z. (2010). Preparation and Electrochemical Capacitance of Cobalt Oxide (Co3O4) Nanotubes as Supercapacitor Material. Electrochim. Acta.

[cit8] Wang Y.-M., Zhang X., Guo C.-Y., Zhao Y.-Q., Xu C.-L., Li H.-L. (2013). Controllable Synthesis of 3D NiχCo1−χ Oxides with Different Morphologies for High-Capacity Supercapacitors. J. Mater. Chem. A.

[cit9] Giannakou P., Masteghin M. G., Slade R. C. T., Hinder S. J., Shkunov M. (2019). Energy Storage on Demand: Ultra-High-Rate and High-Energy-Density Inkjet-Printed NiO Micro-Supercapacitors. J. Mater. Chem. A.

[cit10] Zhang H., Chen Y., Wang W., Zhang G., Zhuo M., Zhang H., Yang T., Li Q., Wang T. (2013). Hierarchical Mo-Decorated Co3O4 Nanowire Arrays on Ni Foam Substrates for Advanced Electrochemical Capacitors. J. Mater. Chem. A.

[cit11] Steinebach H., Kannan S., Rieth L., Solzbacher F. (2010). H2 Gas Sensor Performance of NiO at High Temperatures in Gas Mixtures. Sens. Actuators, B.

[cit12] Hotovy I., Rehacek V., Siciliano P., Capone S., Spiess L. (2002). Sensing Characteristics of NiO Thin Films as NO2 Gas Sensor. Thin Solid Films.

[cit13] Xu J. M., Cheng J. P. (2016). The Advances of Co 3 O 4 as Gas Sensing Materials: A Review. J. Alloys Compd..

[cit14] Shanmugasundaram A., Chinh N. D., Jeong Y.-J., Hou T. F., Kim D.-S., Kim D., Kim Y.-B., Lee D.-W. (2019). Hierarchical Nanohybrids of B- and N-Codoped Graphene/Mesoporous NiO Nanodisks: An Exciting New Material for Selective Sensing of H 2 S at near Ambient Temperature. J. Mater. Chem. A.

[cit15] Varghese B., Reddy M. V., Yanwu Z., Lit C. S., Hoong T. C., Subba Rao G. V., Chowdari B. V. R., Wee A. T. S., Lim C. T., Sow C.-H. (2008). Fabrication of NiO Nanowall Electrodes for High Performance Lithium Ion Battery. Chem. Mater..

[cit16] Kang Y.-M., Song M.-S., Kim J.-H., Kim H.-S., Park M.-S., Lee J.-Y., Liu H. K., Dou S. X. (2005). A Study on the Charge–Discharge Mechanism of Co3O4 as an Anode for the Li Ion Secondary Battery. Electrochim. Acta.

[cit17] Chou S.-L., Wang J.-Z., Liu H.-K., Dou S.-X. (2008). Electrochemical Deposition of Porous Co3O4 Nanostructured Thin Film for Lithium-Ion Battery. J. Power Sources.

[cit18] Rehman A.-U., Iftikhar M., Latif S., Jevtovic V., Ashraf I. M., El-Zahhar A. A., Musad Saleh E. A., Mustansar Abbas S. (2022). Current Advances and Prospects in NiO-Based Lithium-Ion Battery Anodes. Sustainable Energy Technol. Assess..

[cit19] Liu Y., Zhang X. (2009). Effect of Calcination Temperature on the Morphology and Electrochemical Properties of Co3O4 for Lithium-Ion Battery. Electrochim. Acta.

[cit20] Cheng C.-F., Chen Y.-M., Zou F., Yang K.-C., Lin T.-Y., Liu K., Lai C.-H., Ho R.-M., Zhu Y. (2018). Nanoporous Gyroid Ni/NiO/C Nanocomposites from Block Copolymer Templates with High Capacity and Stability for Lithium Storage. J. Mater. Chem. A.

[cit21] Zhu Y., Guo H., Wu Y., Cao C., Tao S., Wu Z. (2014). Surface-Enabled Superior Lithium Storage of High-Quality Ultrathin NiO Nanosheets. J. Mater. Chem. A.

[cit22] Hao W., Chen S., Cai Y., Zhang L., Li Z., Zhang S. (2014). Three-Dimensional Hierarchical Pompon-like Co3O4 Porous Spheres for High-Performance Lithium-Ion Batteries. J. Mater. Chem. A.

[cit23] Xu X., Hou X., Du P., Zhang C., Zhang S., Wang H., Toghan A., Huang M. (2022). Controllable Ni/NiO Interface Engineering on N-Doped Carbon Spheres for Boosted Alkaline Water-to-Hydrogen Conversion by Urea Electrolysis. Nano Res..

[cit24] Xiao J., Zhang X., Gao T., Zhou C., Xiao D. (2017). Electrochemical Formation of Multilayered NiO Film/Ni Foam as a High-Efficient Anode for Methanol Electrolysis. J. Solid State Electrochem..

[cit25] Ma G., Zhang X., Zhou G., Wang X. (2021). Hydrogen Production from Methanol Reforming Electrolysis at NiO Nanosheets Supported Pt Nanoparticles. Chem. Eng. J..

[cit26] van Limpt R. T. M., Lao M., Tsampas M. N., Creatore M. (2024). Unraveling the Role of the Stoichiometry of Atomic Layer Deposited Nickel Cobalt Oxides on the Oxygen Evolution Reaction. Adv. Sci..

[cit27] Haghverdi Khamene S., van Helvoirt C., Tsampas M. N., Creatore M. (2023). Electrochemical Activation of Atomic-Layer-Deposited Nickel Oxide for
Water Oxidation. J. Phys. Chem. C.

[cit28] Dutta R., Maity A., Marsicano A., Ceretti M., Chernyshov D., Bosak A., Villesuzanne A., Roth G., Perversi G., Paulus W. (2020). Long-Range Oxygen Ordering Linked to Topotactic Oxygen Release in Pr 2 NiO 4+*δ* Fuel Cell Cathode Material. J. Mater. Chem. A.

[cit29] Dai W., Bai X., Zhu Y. A., Zhang Y., Lu T., Pan Y., Wang J. (2021). Surface Reconstruction Inducedin Situphosphorus Doping in Nickel Oxides for an Enhanced Oxygen Evolution Reaction. J. Mater. Chem. A.

[cit30] Zhang C., Zheng F., Zhang Z., Xiang D., Cheng C., Zhuang Z., Li P., Li X., Chen W. (2019). Fabrication of Hollow Pompon-like Co 3 O 4 Nanostructures with Rich Defects and High-Index Facet Exposure for Enhanced Oxygen Evolution Catalysis. J. Mater. Chem. A.

[cit31] Sun H., Ang H. M., Tadé M. O., Wang S. (2013). Co3O4 Nanocrystals with Predominantly Exposed Facets: Synthesis, Environmental and Energy Applications. J. Mater. Chem. A.

[cit32] Li X., Su X., Pei Y., Liu J., Zheng X., Tang K., Guan G., Hao X. (2019). Generation of Edge Dislocation Defects in Co 3 O 4 Catalysts: An Efficient Tactic to Improve Catalytic Activity for Oxygen Evolution. J. Mater. Chem. A.

[cit33] Tong S., Zheng M., Lu Y., Lin Z., Li J., Zhang X., Shi Y., He P., Zhou H. (2015). Mesoporous NiO with a Single-Crystalline Structure Utilized as a Noble Metal-Free Catalyst for Non-Aqueous Li-O2 Batteries. J. Mater. Chem. A.

[cit34] Kim C. W., Son Y. S., Pawar A. U., Kang M. J., Zheng J. Y., Sharma V., Mohanty P., Kang Y. S. (2014). Facile Fabrication and Photoelectrochemical Properties of a One Axis-Oriented NiO Thin Film with a (111) Dominant Facet. J. Mater. Chem. A.

[cit35] Wu H., Geng J., Han P., Ge H., Alenizi A. M., Zheng G. (2017). Unconventional Mesoporous Single Crystalline NiO by Synergistically Controlled Evaporation and Hydrolysis. J. Mater. Chem. A.

[cit36] Zheng M., Dong H., Xiao Y., Hu H., He C., Liang Y., Lei B., Sun L., Liu Y. (2017). Hierarchical NiO Mesocrystals with Tuneable High-Energy Facets for Pseudocapacitive Charge Storage. J. Mater. Chem. A.

[cit37] Bao W., Wang R., Li B., Qian C., Zhang Z., Li J., Liu F. (2021). Stable Alkali Metal Anodes Enabled by Crystallographic Optimization - a Review. J. Mater. Chem. A.

[cit38] Hermawan A., Hanindriyo A. T., Hongo K., Maezono R., Yin S. (2022). Impact of Surface Faceting on Gas Sensing Selectivity of NiO: Revealing the Adsorption Sites of Organic Vapors on the {111} Facet. J. Phys. Chem. C.

[cit39] Yu T., Li Z., Zheng H., Chen L., Song W., Zhao Z., Li J., Liu J. (2019). The Nature of Ni-O Pairs for Ethane Activation on NiO(100) and NiO(110) Surfaces. Mol. Catal..

[cit40] Gao R., Zhu J., Xiao X., Hu Z., Liu J., Liu X. (2015). Facet-Dependent Electrocatalytic Performance of Co3O4 for Rechargeable Li-O2 Battery. J. Phys. Chem. C.

[cit41] Zeng Y., Li T., Zhong J., Mao H., Fu M., Ye D., Hu Y. (2023). Unraveling the Role of Co3O4 Facet for Photothermal Catalytic Oxidation of Methanol *via* Operando Spectroscopy and Theoretical Investigation. J. Colloid Interface Sci..

[cit42] Liu Z., Amin H. M. A., Peng Y., Corva M., Pentcheva R., Tschulik K. (2023). Facet-Dependent Intrinsic Activity of Single Co3O4 Nanoparticles for Oxygen Evolution Reaction. Adv. Funct. Mater..

[cit43] Davis E. M., Bergmann A., Kuhlenbeck H., Roldan Cuenya B. (2024). Facet Dependence of the Oxygen Evolution Reaction on Co3O4, CoFe2O4, and Fe3O4 Epitaxial Film Electrocatalysts. J. Am. Chem. Soc..

[cit44] Wiegmann T., Pacheco I., Reikowski F., Stettner J., Qiu C., Bouvier M., Bertram M., Faisal F., Brummel O., Libuda J., Drnec J., Allongue P., Maroun F., Magnussen O. M. (2022). Operando Identification of the Reversible Skin Layer on Co 3 O 4 as a Three-Dimensional Reaction Zone for Oxygen Evolution. ACS Catal..

[cit45] Poulain R., Klein A., Proost J. (2018). Electrocatalytic Properties of (100)-, (110)-, and (111)-Oriented NiO Thin Films toward the Oxygen Evolution Reaction. J. Phys. Chem. C.

[cit46] Burriel M., Garcia G., Santiso J., Abrutis A., Saltyte Z., Figueras A. (2005). Growth Kinetics, Composition, and Morphology of Co3O4 Thin Films Prepared by Pulsed Liquid-Injection MOCVD. Chem. Vap. Deposition.

[cit47] Burriel M., Garcia G., Santiso J., Hansson A. N., Linderoth S., Figueras A. (2005). Co3O4 Protective Coatings Prepared by Pulsed Injection Metal Organic Chemical Vapour Deposition. Thin Solid Films.

[cit48] Ferreira F. F., Tabacniks M. H., Fantini M. C. A., Faria I. C., Gorenstein A. (1996). Electrochromic Nickel Oxide Thin Films Deposited under Different Sputtering Conditions. Solid State Ionics.

[cit49] Han S.-Y., Lee D.-H., Chang Y.-J., Ryu S.-O., Lee T.-J., Chang C.-H. (2006). The Growth Mechanism of Nickel Oxide Thin Films by Room-Temperature Chemical Bath Deposition. J. Electrochem. Soc..

[cit50] Raut B. T., Pawar S. G., Chougule M. A., Sen S., Patil V. B. (2011). New Process for Synthesis of Nickel Oxide Thin Films and Their Characterization. J. Alloys Compd..

[cit51] Chen H. L., Lu Y. M., Hwang W. S. (2005). Characterization of Sputtered NiO Thin Films. Surf. Coatings Technol..

[cit52] Berkat L., Cattin L., Reguig A., Regragui M., Bernède J. C. (2005). Comparison of the Physico-Chemical Properties of NiO Thin Films Deposited by Chemical Bath Deposition and by Spray Pyrolysis. Mater. Chem. Phys..

[cit53] Shinde V. R., Mahadik S. B., Gujar T. P., Lokhande C. D. (2006). Supercapacitive Cobalt Oxide (Co3O4) Thin Films by Spray Pyrolysis. Appl. Surf. Sci..

[cit54] Kadam L. D., Patil P. S. (2001). Thickness-Dependent Properties of Sprayed Cobalt Oxide Thin Films. Mater. Chem. Phys..

[cit55] Shalini K., Mane A. U., Shivashankar S. A., Rajeswari M., Choopun S. (2001). Epitaxial Growth of Co3O4 Films by Low Temperature, Low Pressure Chemical Vapour Deposition. J. Cryst. Growth.

[cit56] Apátiga L. M., Castaño V. M. (2006). Magnetic Behavior of Cobalt Oxide Films Prepared by Pulsed Liquid Injection Chemical Vapor Deposition from a Metal-Organic Precursor. Thin Solid Films.

[cit57] Rooth M., Lindahl E., Hårsta A. (2006). Atomic Layer Deposition of Co3O4 Thin Films Using a CoI2/O2 Precursor Combination. Chem. Vap. Deposition.

[cit58] Nalage S. R., Chougule M. A., Sen S., Joshi P. B., Patil V. B. (2012). Sol-Gel Synthesis of Nickel Oxide Thin Films and Their Characterization. Thin Solid Films.

[cit59] Al-Ghamdi A. A., Mahmoud W. E., Yaghmour S. J., Al-Marzouki F. M. (2009). Structure and Optical Properties of Nanocrystalline NiO Thin Film Synthesized by Sol-Gel Spin-Coating Method. J. Alloys Compd..

[cit60] Kang J. K., Rhee S. W. (2001). Chemical Vapor Deposition of Nickel Oxide Films from Ni(C5H5)2/O2. Thin Solid Films.

[cit61] Attri R., Panda D. P., Ghatak J., Rao C. N. R. (2023). High Crystalline Epitaxial Thin Films of NiO by Plasma-Enhanced ALD and Their Properties. APL Mater..

[cit62] Wang Y., Ghanbaja J., Boulet P., Horwat D., Pierson J. F. (2019). Growth, Interfacial Microstructure and Optical Properties of NiO Thin Films with Various Types of Texture. Acta Mater..

[cit63] Lindahl E., Lu J., Ottosson M., Carlsson J. O. (2009). Epitaxial NiO (100) and NiO (111) Films Grown by Atomic Layer Deposition. J. Cryst. Growth.

[cit64] Klepper K. B., Nilsen O., Fjellvåg H. (2007). Epitaxial Growth of Cobalt Oxide by Atomic Layer Deposition. J. Cryst. Growth.

[cit65] Chen H. G., Wang H. S., Jian S. R., Yeh T. L., Feng J. Y. (2023). Epitaxial Growth of Cobalt Oxide Thin Films on Sapphire Substrates Using Atmospheric Pressure Mist Chemical Vapor Deposition. Coatings.

[cit66] Vaz C. A. F., Henrich V. E., Ahn C. H., Altman E. I. (2009). Growth and Characterization of Thin Epitaxial Co3O4 (111) Films. J. Cryst. Growth.

[cit67] Mane A. U., Shalini K., Wohlfart A., Devi A., Shivashankar S. A. (2002). Strongly Oriented Thin Films of Co3O4 Deposited on Single-Crystal MgO(100) by Low-Pressure, Low-Temperature MOCVD. J. Cryst. Growth.

[cit68] Battiato S., Giangregorio M. M., Catalano M. R., Lo Nigro R., Losurdo M., Malandrino G. (2016). Morphology-Controlled Synthesis of NiO Films: The Role of the Precursor and the Effect of the Substrate Nature on the Films’ Structural/Optical Properties. RSC Adv..

[cit69] Molaei R., Bayati R., Narayan J. (2013). Crystallographic Characteristics and P-Type to n-Type Transition in Epitaxial Nio Thin Film. Cryst. Growth Des..

[cit70] Yang P., Li L., Yu S., Zheng H., Peng W. (2019). The Annealing Temperature and Films Thickness Effect on the Surface Morphology, Preferential Orientation and Dielectric Property of NiO Films. Appl. Surf. Sci..

[cit71] Sasaki S., Fujino K., Takéuchi Y. (1979). X-Ray, Determination of Electron-Density Distributions in Oxides, MgO, MnO, CoO, and NiO, and Atomic Scattering Factors of Their Constituent Atoms. Proc. Jpn. Acad., Ser. B.

[cit72] Picard J. P., Baud G., Besse J. P., Chevalier R. (1980). Croissance Cristalline et Étude Structurale de Co3O4. J. Less-Common Met..

[cit73] Zhang J., Qian J., Ran J., Xi P., Yang L., Gao D. (2020). Engineering Lower Coordination Atoms onto NiO/Co3O4 Heterointerfaces for Boosting Oxygen Evolution Reactions. ACS Catal..

[cit74] Lou C., Pan H., Mei H., Lu G., Liu X., Zhang J. (2022). Low Coordination States in Co3O4/NiOx Heterostructures by Atomic Layer Deposition for Enhanced Gas Detection. Chem. Eng. J..

[cit75] Adhikari S., Selvaraj S., Ji S. H., Kim D. H. (2020). Encapsulation of Co3O4 Nanocone Arrays *via* Ultrathin NiO for Superior Performance Asymmetric Supercapacitors. Small.

[cit76] Nam D., Kim J. (2022). Development of NiO/Co3O4 Nanohybrids Catalyst with Oxygen Vacancy for Oxygen Evolution Reaction Enhancement in Alkaline Solution. Int. J. Hydrogen Energy.

[cit77] Mugheri A. Q., Tahira A., Aftab U., Abro M. I., Chaudhry S. R., Amaral L., Ibupoto Z. H. (2019). Co3O4/NiO Bifunctional Electrocatalyst for Water Splitting. Electrochim. Acta.

[cit78] Wang Y., Kong M., Liu Z., Lin C., Zeng Y. (2017). Morella-Rubra -like Metal–Organic-Framework-Derived Multilayered Co3O4 /NiO/C Hybrids as High-Performance Anodes for Lithium Storage. J. Mater. Chem. A.

[cit79] Xu K., Zou R., Li W., Xue Y., Song G., Liu Q., Liu X., Hu J. (2013). Self-Assembling Hybrid NiO/Co3O4 Ultrathin and Mesoporous Nanosheets into Flower-like Architectures for Pseudocapacitance. J. Mater. Chem. A.

[cit80] Yin X., Zhi C., Sun W., Lv L.-P., Wang Y. (2019). Multilayer NiO@Co3O4 @graphene Quantum Dots Hollow Spheres for High-Performance Lithium-Ion Batteries and Supercapacitors. J. Mater. Chem. A.

[cit81] Stevens M. B., Enman L. J., Korkus E. H., Zaffran J., Trang C. D. M., Asbury J., Kast M. G., Toroker M. C., Boettcher S. W. (2019). Ternary Ni-Co-Fe Oxyhydroxide Oxygen Evolution Catalysts: Intrinsic Activity Trends, Electrical Conductivity, and Electronic Band Structure. Nano Res..

[cit82] Ou Y., Twight L. P., Samanta B., Liu L., Biswas S., Fehrs J. L., Sagui N. A., Villalobos J., Morales-Santelices J., Antipin D., Risch M., Toroker M. C., Boettcher S. W. (2023). Cooperative Fe Sites on Transition Metal (Oxy)Hydroxides Drive High Oxygen Evolution Activity in Base. Nat. Commun..

[cit83] Zhang L., Xu Q., Hu Y., Chen L., Jiang H. (2023). Benchmarking the PH-Stability Relationship of Metal Oxide Anodes in Anion Exchange Membrane Water Electrolysis. ACS Sustainable Chem. Eng..

[cit84] Natarajan K., Munirathinam E., Yang T. C. K. (2021). Operando Investigation of Structural and Chemical Origin of Co3O4Stability in Acid under Oxygen Evolution Reaction. ACS Appl. Mater. Interfaces.

[cit85] Chung T. M., Lee S. S., Cho W., Kim M., Lee Y. K., Hwang J. H., An K. S., Kim C. G. (2011). Volatile Nickel Aminoalkoxide Complexes as Liquid Precursors for Non-Volatile Memory Device of NiO Films by ALD. Bull. Korean Chem. Soc..

[cit86] Donders M. E., Knoops H. C. M., Kessels W. M. M., Notten P. H. L. (2012). Co3O4 as Anode Material for Thin Film Micro-Batteries Prepared by Remote Plasma Atomic Layer Deposition. J. Power Sources.

[cit87] Nandi D. K., Manna J., Dhara A., Sharma P., Sarkar S. K. (2016). Atomic Layer Deposited Cobalt Oxide: An Efficient Catalyst for NaBH4 Hydrolysis. J. Vac. Sci. Technol., A.

[cit88] Donders M. E., Knoops H. C. M., Van M. C. M., Kessels W. M. M., Notten P. H. L. (2011). Remote Plasma Atomic Layer Deposition of Co3O4 Thin Films. J. Electrochem. Soc..

[cit89] Miikkulainen V., Leskelä M., Ritala M., Puurunen R. L. (2013). Crystallinity of Inorganic Films Grown by Atomic Layer Deposition: Overview and General Trends. J. Appl. Phys..

[cit90] van Limpt R. T. M., Lavorenti M., Verheijen M. A., Tsampas M. N., Creatore M. (2023). Control by Atomic Layer Deposition over the Chemical Composition of Nickel Cobalt Oxide for the Oxygen Evolution Reaction. J. Vac. Sci. Technol., A.

[cit91] Grosvenor A. P., Biesinger M. C., Smart R. S. C., McIntyre N. S. (2006). New Interpretations of XPS Spectra of Nickel Metal and Oxides. Surf. Sci..

[cit92] Biesinger M. C., Payne B. P., Lau L. W. M., Gerson A., Smart R. S. C. (2009). X–ray Photoelectron Spectroscopic Chemical State Quantification of Mixed Nickel Metal, Oxide and Hydroxide Systems. Surf. Interface Anal..

[cit93] McIntyre N. S., Cook M. G. (1975). X-Ray, Photoelectron Studies on Some Oxides and Hydroxides of Cobalt, Nickel, and Copper. Anal. Chem..

[cit94] Mitton D. B., Walton J., Thompson G. E. (1993). An XPS and AES Study of the Ageing of a Co–20%Ni Metal–evaporated Tape. Surf. Interface Anal..

[cit95] Biesinger M. C., Payne B. P., Grosvenor A. P., Lau L. W. M., Gerson A. R., Smart R. S. C. (2011). Resolving Surface Chemical States in XPS Analysis of First Row Transition Metals, Oxides and Hydroxides: Cr, Mn, Fe, Co and Ni. Appl. Surf. Sci..

[cit96] Cabrera–German D., Gomez–Sosa G., Herrera–Gomez A. (2016). Accurate Peak Fitting and Subsequent Quantitative Composition Analysis of the Spectrum of Co2p Obtained with Al Kα Radiation: I: Cobalt Spinel. Surf. Interface Anal..

[cit97] Thompson C. V., Carel R. (1995). Texture Development in Polycrystalline Thin Films. Mater. Sci. Eng., B.

[cit98] Cappus D., Xu C., Ehrlich D., Dillmann B., Ventrice C. A., Al Shamery K., Kuhlenbeck H., Freund H. J. (1993). Hydroxyl Groups on Oxide Surfaces: NiO(100), NiO(111) and Cr2O3(111). Chem. Phys..

[cit99] Profijt H. B., Kudlacek P., van de Sanden M. C. M., Kessels W. M. M. (2011). Ion and Photon Surface Interaction during Remote Plasma ALD of Metal Oxides. J. Electrochem. Soc..

[cit100] Profijt H. B., Potts S. E., van de Sanden M. C. M., Kessels W. M. M. (2011). Plasma-Assisted Atomic Layer Deposition: Basics, Opportunities, and Challenges. J. Vac. Sci. Technol., A.

[cit101] Zhao R., Xiao S., Yang S., Wang X. (2019). Surface Thermolytic Behavior of Nickel Amidinate and Its Implication on the Atomic Layer Deposition of Nickel Compounds. Chem. Mater..

[cit102] Hercules D. M. (1973). Electron Spectroscopy for Chemical Analysis. Proc. Soc. Anal. Chem..

[cit103] Mukherjee K., Kreugel D., Phung N., van Helvoirt C., Zardetto V., Creatore M. (2024). On the VOC Loss in NiO-Based Inverted Metal Halide Perovskite Solar Cells. Mater. Adv..

[cit104] Heil S. B. S., Langereis E., Roozeboom F., van de Sanden M. C. M., Kessels W. M. M. (2006). Low-Temperature Deposition of TiN by Plasma-Assisted Atomic Layer Deposition. J. Electrochem. Soc..

[cit105] Holden K. E. K., Conley J. F. (2019). Characterization of Atomic Layer Deposited Semiconducting Co3O4. J. Vac. Sci. Technol., A.

[cit106] Maxwell J. A., Campbell J. L., Teesdale W. J. (1989). The Guelph PIXE Software Package. Nucl. Instrum. Methods Phys. Res., Sect. B.

[cit107] Barradas N. P., Jeynes C. (2008). Advanced Physics and Algorithms in the IBA DataFurnace. Nucl. Instrum. Methods Phys. Res., Sect. B.

