# Supplementary material for: On the epitaxial growth in ALD Co3O4- and NiO-based bilayers
Source: Nanoscale. 2025 Apr 14;17(17):11037–48. doi: 10.1039/d5nr01212k (PMC11995145; doi:10.1039/d5nr01212k)
Supplement: NR-017-D5NR01212K-s001 [file NR-017-D5NR01212K-s001.pdf]

Supplementary Information (SI) for Nanoscale.
This journal is © The Royal Society of Chemistry 2025

Supporting Information

On the epitaxial growth in ALD Co_3O_4- and NiO-based bilayers

Renée T.M. van Limpt,*[a] Cristian A. A. van Helvoirt,[a] Mariadriana Creatore[a,b] and Marcel A. Verheijen*[a,c]

Table S1 Information on the crystal structure of NiO (ICSD 9866) and Co_3O_4 (ICSD 36256).

	**NiO**	**Co_3O_4**
Crystal system	Cubic	Cubic
Space group	Fm3m (225)	Fd3m (227)
Z	4	8
Lattice constant	4.1780 Å	8.0720 Å
Interatomic distance (Ni-Ni / Co-Co)	2.954 Å	2.854 Å
Interplanar spacing (d_{xyz})	d_{100} = 4.178 Å<br>d_{110} = 2.954 Å<br>d_{111} = 2.412 Å	d_{200} = 4.036 Å<br>d_{220} = 2.854 Å<br>d_{333} = 2.330 Å

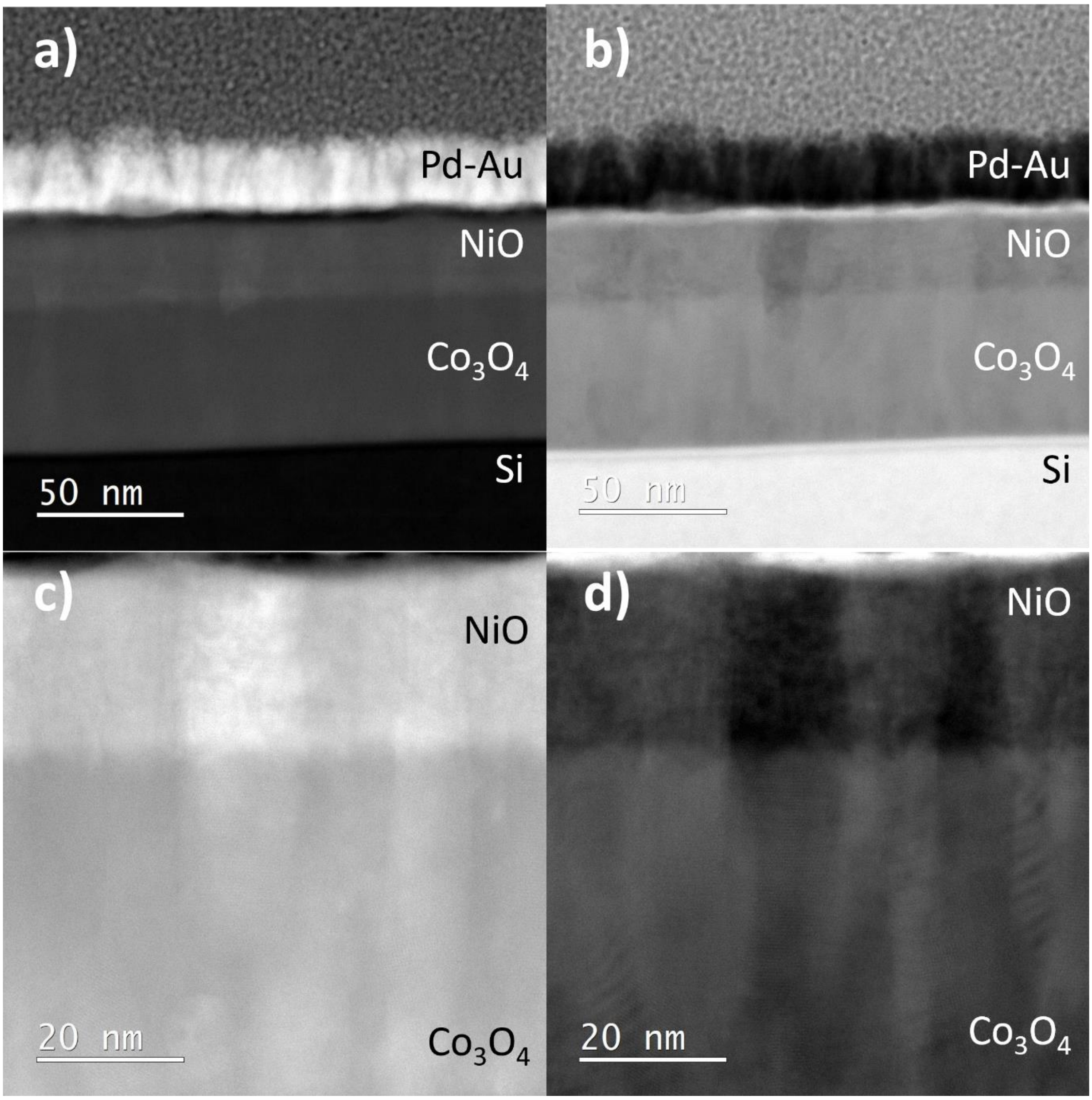

Figure S1 Complementary (a,c) High Angle Annular Dark Field (HAADF)-STEM and (b,d) Bright Field (BF)-STEM images of the c-Si/53nm Co_3O_4/27nm NiO stack.

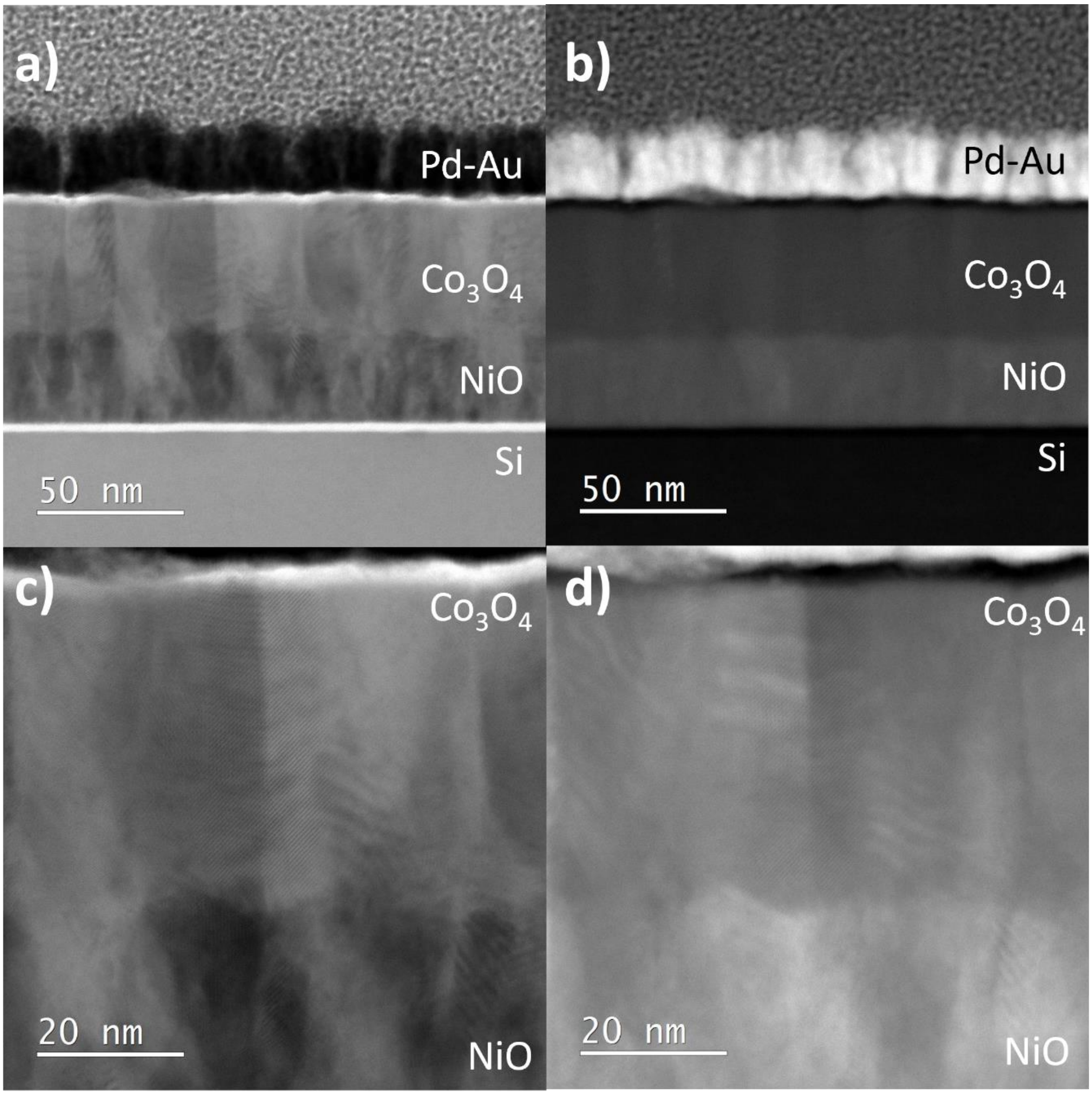

Figure S2 Complementary (a,c) BF-STEM and (b,d) HAADF-STEM images of c-Si/29 nm NiO/42 nm Co_3O_4 stack

Film texture

As discussed in the main text, all poly-crystalline films in our studies are textured, i.e. the crystals in these films grow along a preferential crystallographic orientation. The sharpness of the texture, i.e. the distribution of crystallite orientations in the film has been primarily studied by rocking curve XRD measurements. In a rocking curve[1], the Bragg angle, 2θ, is fixed to a selected reflection in the $2\theta/\omega$ scan, typically the peak characteristic for the texture, and the sample is tilted (or 'rocked') in the ω axis such that planes no longer parallel with the sample surface are brought onto the Bragg plane. Typically, for rocking curves the tilt range is a few degrees. However, thin-film polycrystalline films may have a broader distribution.

The downside of analysing a broad angular distribution is the broad range of the incidence angle ω, which may lead to a change in probed volume as a function of ω, thereby affecting the peak intensity independently of a possible texture. To reduce this effect, studies have been limited to positive values of $\Delta\omega$. As an independent comparison of these texture studies, electron diffraction has been adopted for one sample, as discussed in more detail below. Rocking curves have been acquired for 3 sets of samples.

Texture in Co_3O_4 thin films.

First, Co_3O_4 films with two different thicknesses have been studied. The intensity of the (2 2 2) peak of the Co_3O_4 film is measured for both the 51nm-thick Co_3O_4 film presented in the manuscript and an additional 80 nm-thick Co_3O_4 film (**Figure S3a**). These measurements reveal a FWHM of the (2 2 2) peak of ~6° for the 51 nm-thick film and of ~4° for the 80 nm-thick film, highlighting the broad distribution of crystallite orientations characteristic for polycrystalline films. The slightly smaller FWHM for the thickest film suggests only a slight narrowing of its texture.

Complementary electron diffraction measurements were performed on a 26 nm-thick Co_3O_4 film deposited on an electron transparent TEM window. Where XRD studies provide information on the horizontal planes, electrons in a TEM geometry diffract at vertically oriented lattice planes. As an example, in case of a sharp Co_3O_4 <1 1 1> texture, a strong (1 1 1) peak will be visible in XRD gonio plots, and all other peaks will be absent. Alternatively , in electron diffraction patterns, only lattice planes orthogonal to the (1 1 1) planes will diffract, i.e. (1 -1 0), (2 -1 -1), (3 -2 -1), ..., while the (1 1 1) ring will be absent in electron diffraction patterns taken at normal incidence to a poly-crystalline film.

The pattern in **Figure S4a** shows signs of <1 1 1> texture: the (1 1 1) ring is almost absent, while the (0 2 -2) ring is bright. The fact that a weak (1 1 1) ring is present, suggests that the <1 1 1> texture is not perfect.

Rotation of the film with respect to the electron beam around an in-plane axis will cause planes orthogonal to the tilt axis to remain vertical and therefore contribute to the electron diffraction pattern, while planes parallel to the axis tilt away from the vertical orientation and therefore disappear. This is apparent in **Figure S4b** for the (0 2 2) ring. The intensity ratio between the parallel (P) and orthogonal (O) plane direction can therefore be used to determine the sharpness of the texture. **Figure S3b** displays this ratio for the (0 2 2) reflection, measured as a function of sample tilt in a tilt range between 0-30°, suggesting an angular distribution with a FWHM of ~7°.

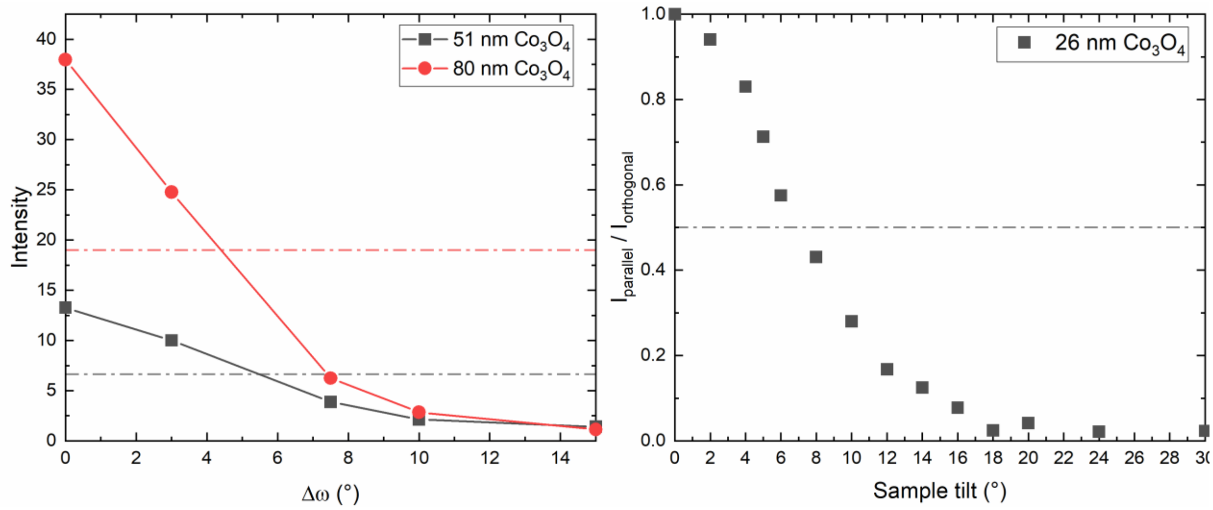

Figure S3 (a) Intensity of the (2 2 2) Co_3O_4 peak as a function of tilt angle obtained using rocking curve XRD measurements for a 51 nm-thick Co_3O_4 and 80 nm-thick Co_3O_4 film. The horizontal lines serve as a guide to the eye for the FWHM of the films. (b) Intensity ratio of the intensity distribution along the (0 2 2) diffraction ring in electron diffraction patterns of a 26 nm-thick Co_3O_4 film as a function of sample tilt, as displayed in Figure S4b.

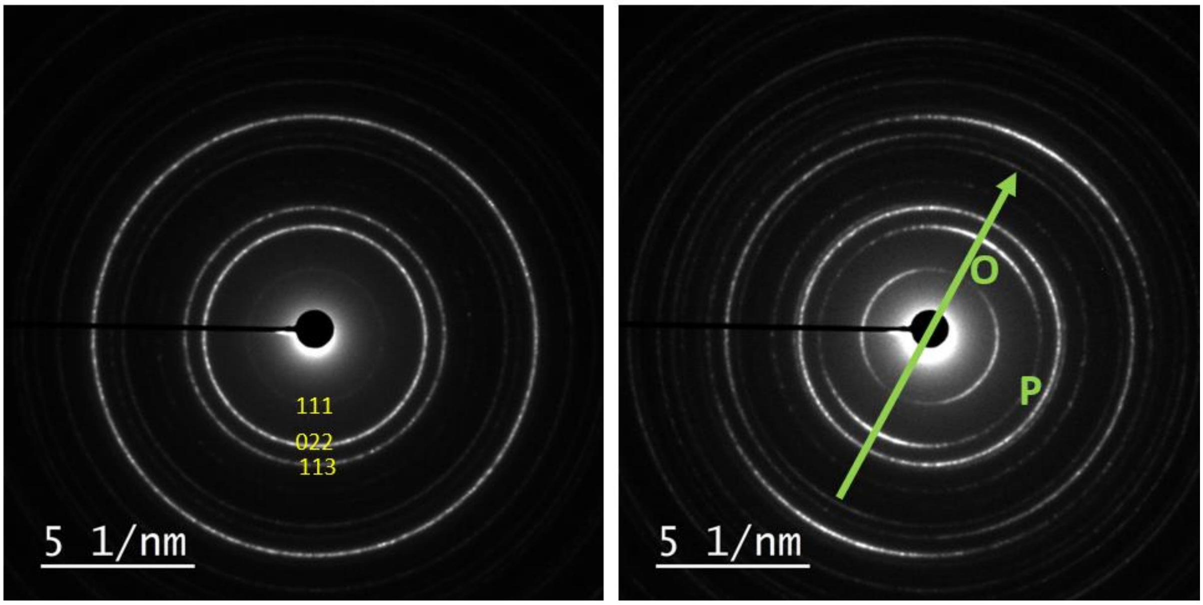

Figure S4 Electron diffraction patterns acquired (a) at normal incidence and (b) at 20° from normal incidence. The (0 2 2) ring has split up into arc segments due to the presence of texture in the film. The Miller indices of the inner 3 rings are indicated. The arrow in figure b indicates the orientation of the sample tilt axis.

Texture in bilayer stacks.

The next two sets of rocking curve studies study bilayer stacks.

The c-Si/29 nm-thick NiO/42 nm-thick Co_3O_4 stack was shown to have a <1 0 0> texture. The rocking curves in **Figure S5a** show the intensity of the (2 0 0)/(4 0 0) peaks as a function of $\Delta\omega$. Similar FWHM values are obtained for both layers in the c-Si/NiO/Co_3O_4 stack at ~11° for the (2 0 0) NiO peak and ~10° for (4 0 0) Co_3O_4 peak.

For the c-Si/53 nm-thick Co_3O_4/27 nm-thick NiO stack the <1 1 1> texture has been studied (**Figure S5b**). Stacks with an underlying Co_3O_4 film show smaller variation in texture distribution, as evidenced by the ~5° variation for the (2 2 2) peak of the Co_3O_4 layer and ~6° variation for the (1 1 1) peak of the subsequent NiO layer. The angular distribution of the Co_3O_4 film in the stack closely aligns with the angle distribution of the Co_3O_4 film presented in **Figure S3**, highlighting that the deposition of a NiO overlayer does not influence the orientational distribution in the underlying film.

Both data sets suggest that the texture distribution is continued across the interface, with a similar distribution in crystal orientation.

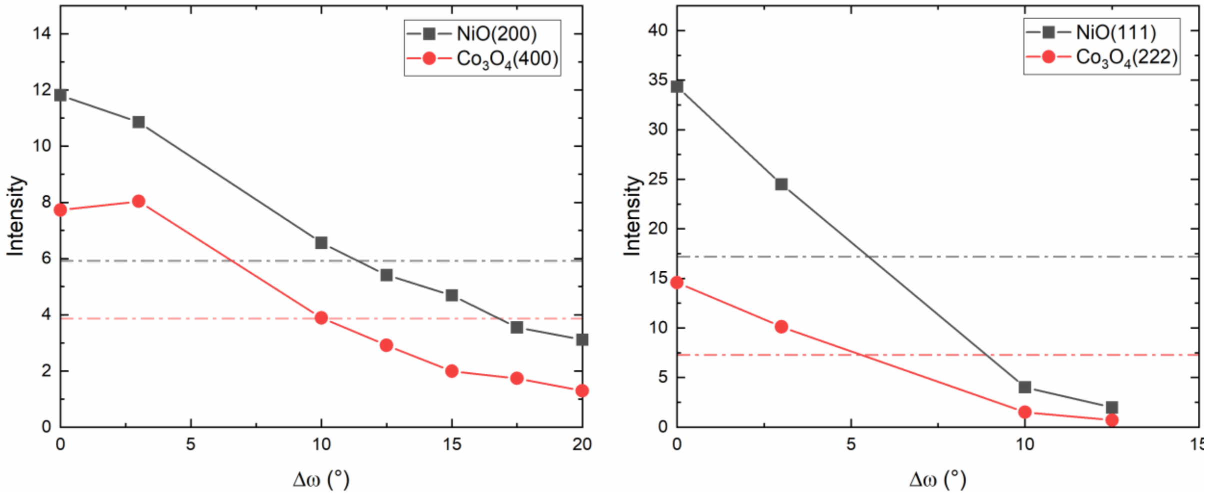

Figure S5 (a) Intensity of the NiO (2 0 0) and Co_3O_4 (4 0 0) peaks of the c-Si/29 nm-thick NiO/42 nm-thick Co_3O_4 stack and (b) NiO (1 1 1) and Co_3O_4 (2 2 2) peaks of the c-Si/53 nm-thick Co_3O_4/27 nm-thick NiO stack as a function of tilt angle obtained using rocking curve XRD measurements. The horizontal lines serve as a guide to the eye for the FWHM of the films.

XPS Auger correction

The overlap of the Ni2p spectrum with Auger features from cobalt oxide and Co2p spectra with Auger features from nickel oxide presents a significant challenge in the interpretation of the spectra, especially for the interpretation of thin film overlayers.[2] Simultaneously, accurate fitting of the 2p features of cobalt and nickel oxide is non-trivial due to the complexity of the spectra. Rather than attempting a direct fit of the overlayer and Auger features, we apply a method that directly subtracts the Auger features from the 2p spectrum.

For clarity, the method is outlined for a cobalt oxide overlayer as it is impacted the most from the Auger feature. However, the same method is applied for a nickel oxide overlayer. First the intensity ratio between the main Ni2p feature and the Auger feature in the Co2p region of a NiO film is calculated (**Figure S6a-b**). Next the film composition of the overlayer is determined using a mathematical fit of the Ni3p and Co3p spectra, using a method outlined previously[3]. Based on this composition, an additional normalisation of the Auger feature is performed (**Figure S6c**). This scaled reference Auger spectrum is subtracted from the Co2p spectrum, effectively removing the overlapping features (**Figure S6d**) without the need for a fitting procedure. The approach is validated using an Mg K_α source, which prevents overlap with the Auger features, and shows the same trend as the corrected Al K_α source.

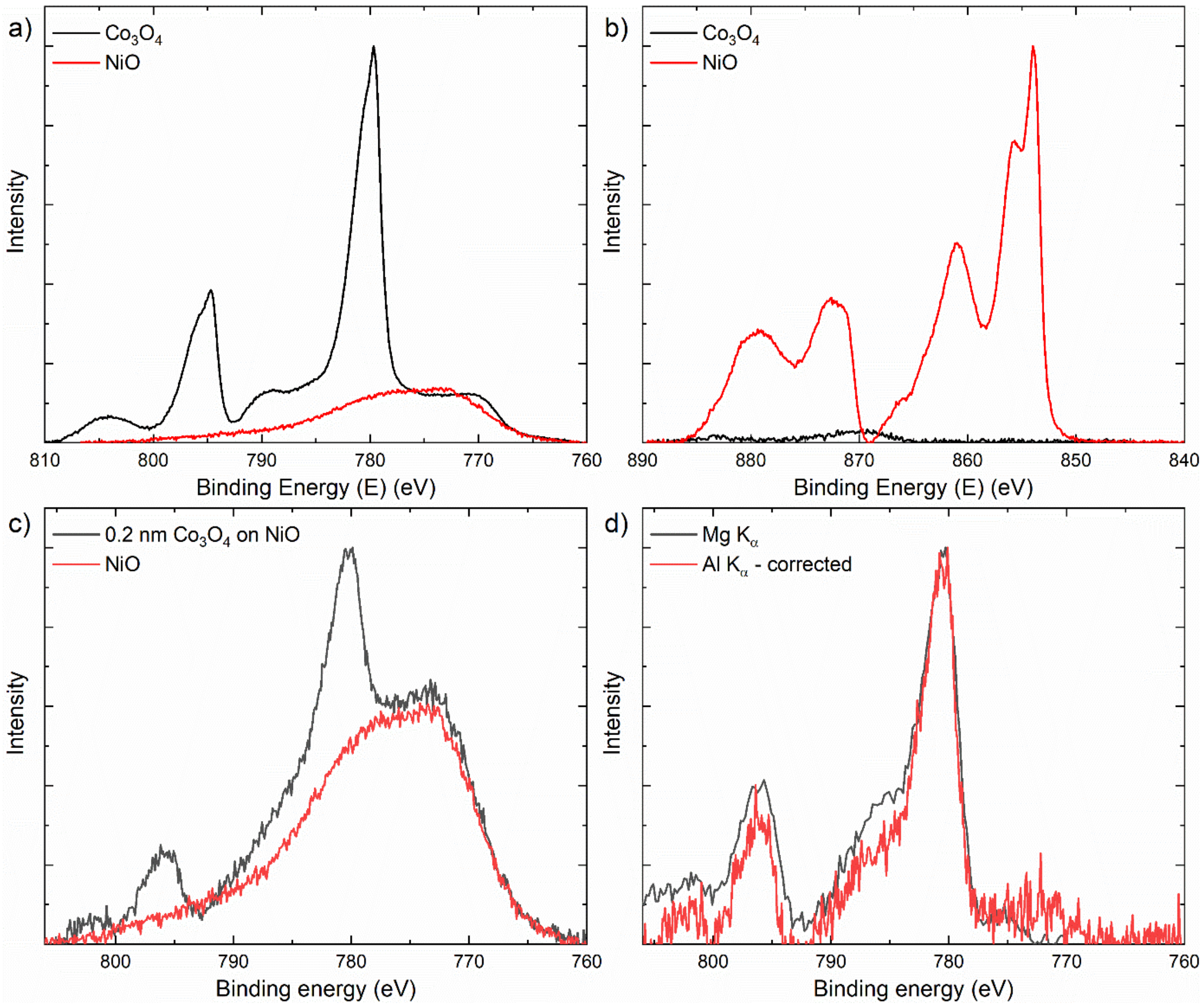

Figure S6 XPS measurement of thick Co_3O_4 and NiO films in (a) the Co2p and (b) the Ni2p spectrum. The Auger features are normalised to their respective 2p features. (c) The Co2p spectrum of the NiO Auger featured scaled to a 0.2 nm Co_3O_4 film on NiO as described previously. The correction procedure is validated in (d) through comparison of the corrected 0.2 nm Co_3O_4 on NiO measurement to a measurement using a Mg K_α source.

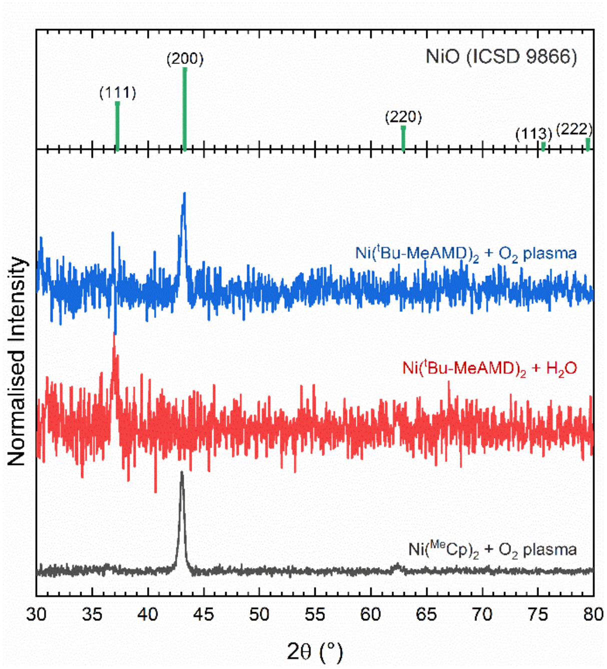

Figure S7 Gonio XRD graphs of NiO films on c-Si using 3 different ALD processes at 150°C substrate table temperature.

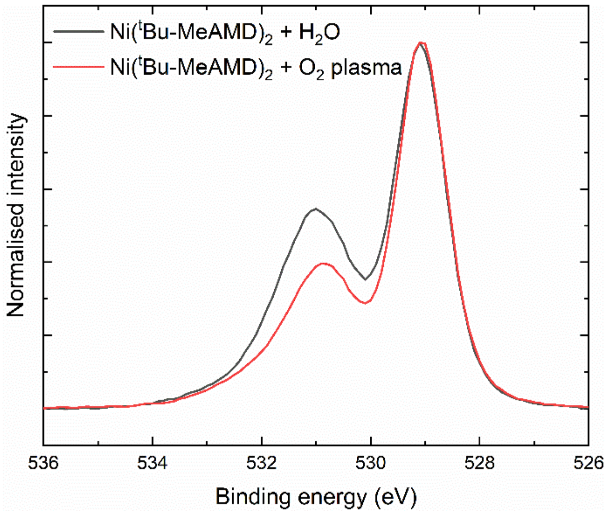

Figure S8 XPS measurement of the O1s spectrum on NiO films based on Ni(^{t}Bu-MeAMD)$_2$ with a H_2O or O_2 plasma co-reactant.

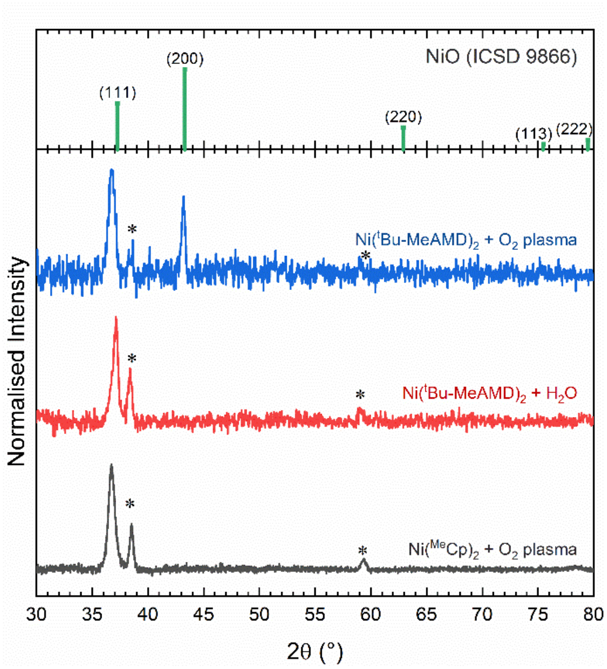

Figure S9 Gonio XRD graphs of NiO films on Co_3O_4 using 3 different ALD processes at 150°C substrate table temperature.

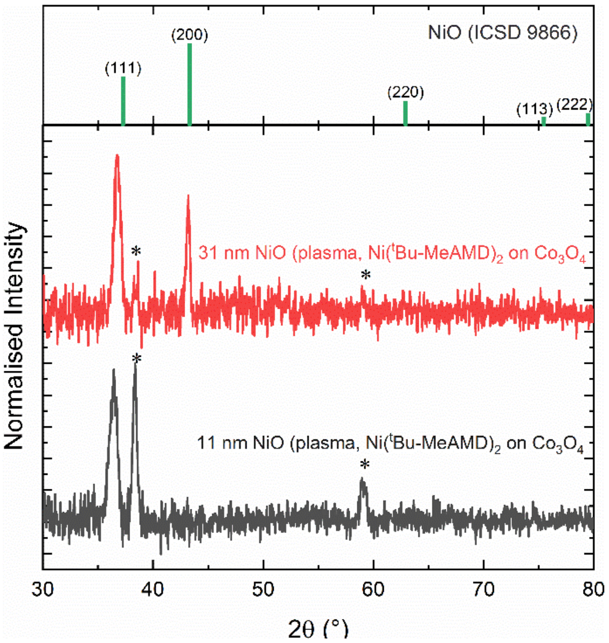

Figure S10 Gonio XRD graphs of two thicknesses of NiO deposited using Ni(^{t}Bu-MeAMD)$_2$ and O_2 plasma on Co_3O_4.

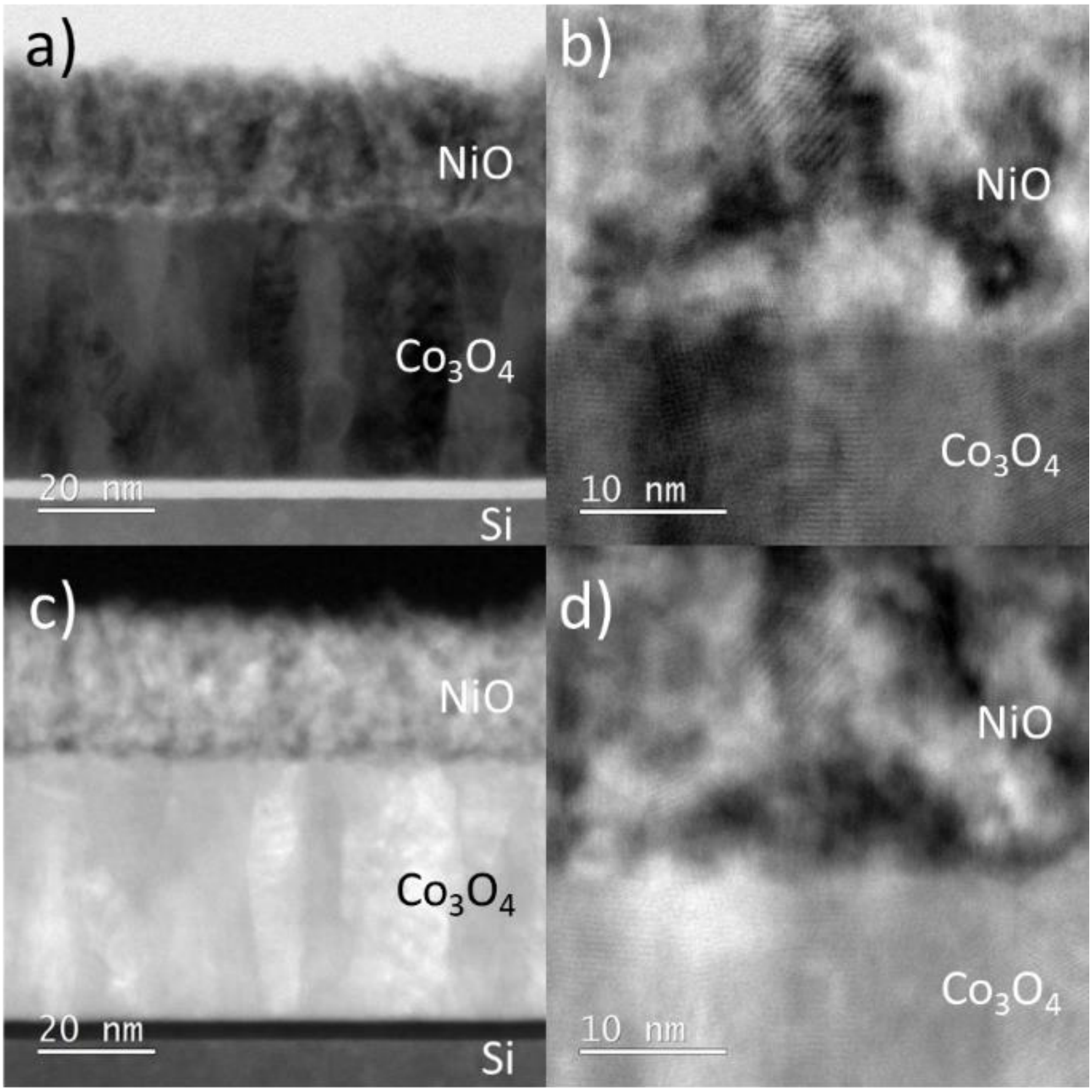

Figure S11 Complementary (a,b) BF-STEM and (c,d) HAADF-STEM images of the NiO/Co_3O_4 stack.

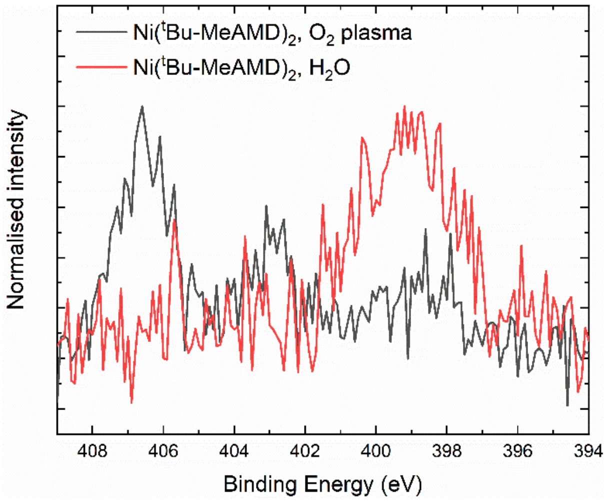

Figure S12 XPS N1s spectra of the two Ni(tBu-MeAMD)$_2$ based NiO films.

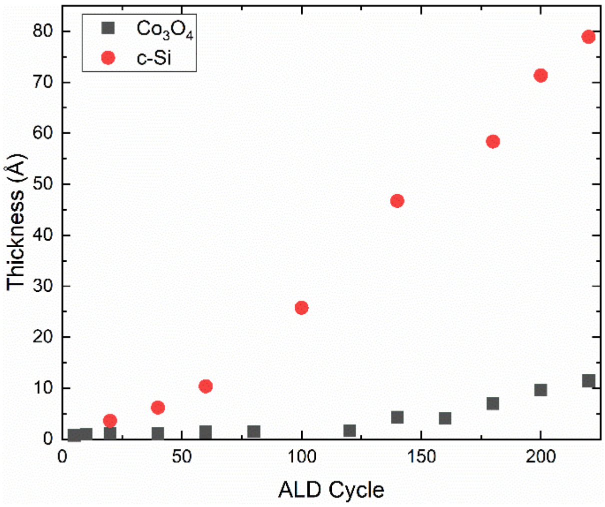

Figure S13 XPS derived thickness of NiO deposited using Ni(tBu-MeAMD)$_2$ and H_2O on c-Si and Co_3O_4.

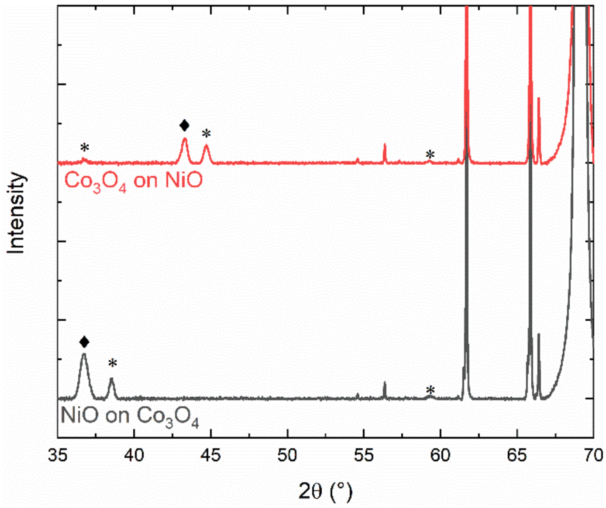

Figure S14 Gonio X-ray diffractograms of the c-Si/NiO/Co_3O_4 and c-Si/Co_3O_4/NiO stacks presented in Figure 1 of the manuscript without the 3° axis offset. Peaks associated with the NiO rock salt structure are indicated by ♦ and peaks associated with the spinel phase of Co_3O_4 by *.

References

(1) Harrington, G. F.; Santiso, J. Back-to-Basics Tutorial: X-Ray Diffraction of Thin Films. *J. Electroceramics* **2021**, *47* (4), 141–163.

(2) van Limpt, R. T. M.; Lavorenti, M.; Verheijen, M. A.; Tsampas, M. N.; Creatore, M. Control by Atomic Layer Deposition over the Chemical Composition of Nickel Cobalt Oxide for the Oxygen Evolution Reaction. *J. Vac. Sci. Technol. A* **2023**, *41* (3), 32407.

(3) van Limpt, R. T. M.; Lao, M.; Tsampas, M. N.; Creatore, M. Unraveling the Role of the Stoichiometry of Atomic Layer Deposited Nickel Cobalt Oxides on the Oxygen Evolution Reaction. *Adv. Sci.* **2024**, *11* (32), 2405188.
